# Search for resonances decaying to an anomalous jet and a Higgs boson in proton–proton collisions at $\sqrt{s}=13\,\text {Te}\hspace{-.08em}\text {V}$

**DOI:** 10.1140/epjc/s10052-025-15176-9

**Published:** 2026-02-10

**Authors:** CMS Collaboration, A. Hayrapetyan, V. Makarenko, A. Tumasyan, W. Adam, J. W. Andrejkovic, L. Benato, T. Bergauer, M. Dragicevic, C. Giordano, P. S. Hussain, M. Jeitler, N. Krammer, A. Li, D. Liko, M. Matthewman, I. Mikulec, J. Schieck, R. Schöfbeck, D. Schwarz, M. Shooshtari, M. Sonawane, W. Waltenberger, C.-E. Wulz, T. Janssen, H. Kwon, D. Ocampo Henao, T. Van Laer, P. Van Mechelen, J. Bierkens, N. Breugelmans, J. D’Hondt, S. Dansana, A. De Moor, M. Delcourt, F. Heyen, Y. Hong, P. Kashko, S. Lowette, I. Makarenko, D. Müller, J. Song, S. Tavernier, M. Tytgat, G. P. Van Onsem, S. Van Putte, D. Vannerom, B. Bilin, B. Clerbaux, A. K. Das, I. De Bruyn, G. De Lentdecker, H. Evard, L. Favart, P. Gianneios, A. Khalilzadeh, F. A. Khan, A. Malara, M. A. Shahzad, L. Thomas, M. Vanden Bemden, C. Vander Velde, P. Vanlaer, F. Zhang, M. De Coen, D. Dobur, G. Gokbulut, J. Knolle, D. Marckx, K. Skovpen, N. Van Den Bossche, J. van der Linden, J. Vandenbroeck, L. Wezenbeek, S. Bein, A. Benecke, A. Bethani, G. Bruno, A. Cappati, J. De Favereau De Jeneret, C. Delaere, A. Giammanco, A. O. Guzel, V. Lemaitre, J. Lidrych, P. Malek, P. Mastrapasqua, S. Turkcapar, G. A. Alves, M. Barroso Ferreira Filho, E. Coelho, C. Hensel, T. Menezes De Oliveira, C. Mora Herrera, P. Rebello Teles, M. Soeiro, E. J. Tonelli Manganote, A. Vilela Pereira, W. L. Aldá Júnior, H. Brandao Malbouisson, W. Carvalho, J. Chinellato, M. Costa Reis, E. M. Da Costa, G. G. Da Silveira, D. De Jesus Damiao, S. Fonseca De Souza, R. Gomes De Souza, S. S. Jesus, T. Laux Kuhn, M. Macedo, K. Mota Amarilo, L. Mundim, H. Nogima, J. P. Pinheiro, A. Santoro, A. Sznajder, M. Thiel, F. Torres Da Silva De Araujo, C. A. Bernardes, F. Damas, T. R. Fernandez Perez Tomei, E. M. Gregores, B. Lopes Da Costa, I. Maietto Silverio, P. G. Mercadante, S. F. Novaes, B. Orzari, Sandra S. Padula, V. Scheurer, A. Aleksandrov, G. Antchev, P. Danev, R. Hadjiiska, P. Iaydjiev, M. Shopova, G. Sultanov, A. Dimitrov, L. Litov, B. Pavlov, P. Petkov, A. Petrov, S. Keshri, D. Laroze, S. Thakur, W. Brooks, T. Cheng, T. Javaid, L. Wang, L. Yuan, Z. Hu, Z. Liang, J. Liu, X. Wang, G. M. Chen, H. S. Chen, M. Chen, Y. Chen, Q. Hou, X. Hou, F. Iemmi, C. H. Jiang, A. Kapoor, H. Liao, G. Liu, Z.-A. Liu, J. N. Song, S. Song, J. Tao, C. Wang, J. Wang, H. Zhang, J. Zhao, A. Agapitos, Y. Ban, A. Carvalho Antunes De Oliveira, S. Deng, B. Guo, Q. Guo, C. Jiang, A. Levin, C. Li, Q. Li, Y. Mao, S. Qian, S. J. Qian, X. Qin, X. Sun, D. Wang, J. Wang, H. Yang, M. Zhang, Y. Zhao, C. Zhou, S. Yang, Z. You, K. Jaffel, N. Lu, G. Bauer, B. Li, H. Wang, K. Yi, J. Zhang, Y. Li, Z. Lin, C. Lu, M. Xiao, C. Avila, D. A. Barbosa Trujillo, A. Cabrera, C. Florez, J. Fraga, J. A. Reyes Vega, C. Rendón, M. Rodriguez, A. A. Ruales Barbosa, J. D. Ruiz Alvarez, N. Godinovic, D. Lelas, A. Sculac, M. Kovac, A. Petkovic, T. Sculac, P. Bargassa, V. Brigljevic, B. K. Chitroda, D. Ferencek, K. Jakovcic, A. Starodumov, T. Susa, A. Attikis, K. Christoforou, A. Hadjiagapiou, C. Leonidou, C. Nicolaou, L. Paizanos, F. Ptochos, P. A. Razis, H. Rykaczewski, H. Saka, A. Stepennov, M. Finger, M. Finger, E. Ayala, E. Carrera Jarrin, A. A. Abdelalim, R. Aly, M. Abdullah Al-Mashad, A. Hussein, H. Mohammed, K. Ehataht, M. Kadastik, T. Lange, C. Nielsen, J. Pata, M. Raidal, N. Seeba, L. Tani, A. Milieva, K. Osterberg, M. Voutilainen, N. Bin Norjoharuddeen, E. Brücken, F. Garcia, P. Inkaew, K. T. S. Kallonen, R. Kumar Verma, T. Lampén, K. Lassila-Perini, B. Lehtela, S. Lehti, T. Lindén, N. R. Mancilla Xinto, M. Myllymäki, M. m. Rantanen, S. Saariokari, N. T. Toikka, J. Tuominiemi, H. Kirschenmann, P. Luukka, H. Petrow, M. Besancon, F. Couderc, M. Dejardin, D. Denegri, P. Devouge, J. L. Faure, F. Ferri, P. Gaigne, S. Ganjour, P. Gras, G. Hamel de Monchenault, M. Kumar, V. Lohezic, J. Malcles, F. Orlandi, L. Portales, S. Ronchi, M. Ö. Sahin, A. Savoy-Navarro, P. Simkina, M. Titov, M. Tornago, F. Beaudette, G. Boldrini, P. Busson, C. Charlot, M. Chiusi, T. D. Cuisset, O. Davignon, A. De Wit, T. Debnath, I. T. Ehle, B. A. Fontana Santos Alves, S. Ghosh, A. Gilbert, R. Granier de Cassagnac, L. Kalipoliti, M. Manoni, M. Nguyen, S. Obraztsov, C. Ochando, R. Salerno, J. B. Sauvan, Y. Sirois, G. Sokmen, L. Urda Gómez, A. Zabi, A. Zghiche, J.-L. Agram, J. Andrea, D. Bloch, J.-M. Brom, E. C. Chabert, C. Collard, G. Coulon, S. Falke, U. Goerlach, R. Haeberle, A.-C. Le Bihan, M. Meena, O. Poncet, G. Saha, P. Vaucelle, A. Di Florio, D. Amram, S. Beauceron, B. Blancon, G. Boudoul, N. Chanon, D. Contardo, P. Depasse, H. El Mamouni, J. Fay, S. Gascon, M. Gouzevitch, C. Greenberg, G. Grenier, B. Ille, E. Jourd’huy, I. B. Laktineh, M. Lethuillier, B. Massoteau, L. Mirabito, A. Purohit, M. Vander Donckt, J. Xiao, I. Bagaturia, I. Lomidze, Z. Tsamalaidze, V. Botta, S. Consuegra Rodríguez, L. Feld, K. Klein, M. Lipinski, D. Meuser, P. Nattland, V. Oppenländer, A. Pauls, D. Pérez Adán, N. Röwert, M. Teroerde, C. Daumann, S. Diekmann, A. Dodonova, N. Eich, D. Eliseev, F. Engelke, J. Erdmann, M. Erdmann, B. Fischer, T. Hebbeker, K. Hoepfner, F. Ivone, A. Jung, N. Kumar, M. Y. Lee, F. Mausolf, M. Merschmeyer, A. Meyer, F. Nowotny, A. Pozdnyakov, W. Redjeb, H. Reithler, U. Sarkar, V. Sarkisovi, A. Schmidt, C. Seth, A. Sharma, J. L. Spah, V. Vaulin, S. Zaleski, M. R. Beckers, C. Dziwok, G. Flügge, N. Hoeflich, T. Kress, A. Nowack, O. Pooth, A. Stahl, A. Zotz, H. Aarup Petersen, A. Abel, M. Aldaya Martin, J. Alimena, S. Amoroso, Y. An, I. Andreev, J. Bach, S. Baxter, M. Bayatmakou, H. Becerril Gonzalez, O. Behnke, A. Belvedere, F. Blekman, K. Borras, A. Campbell, S. Chatterjee, L. X. Coll Saravia, G. Eckerlin, D. Eckstein, E. Gallo, A. Geiser, V. Guglielmi, M. Guthoff, A. Hinzmann, L. Jeppe, M. Kasemann, C. Kleinwort, R. Kogler, M. Komm, D. Krücker, W. Lange, D. Leyva Pernia, K.-Y. Lin, K. Lipka, W. Lohmann, J. Malvaso, R. Mankel, I.-A. Melzer-Pellmann, M. Mendizabal Morentin, A. B. Meyer, G. Milella, K. Moral Figueroa, A. Mussgiller, L. P. Nair, J. Niedziela, A. Nürnberg, J. Park, E. Ranken, A. Raspereza, D. Rastorguev, L. Rygaard, M. Scham, S. Schnake, P. Schütze, C. Schwanenberger, D. Selivanova, K. Sharko, M. Shchedrolosiev, D. Stafford, M. Torkian, F. Vazzoler, A. Ventura Barroso, R. Walsh, D. Wang, Q. Wang, K. Wichmann, L. Wiens, C. Wissing, Y. Yang, S. Zakharov, A. Zimermmane Castro Santos, A. R. Alves Andrade, M. Antonello, S. Bollweg, M. Bonanomi, K. El Morabit, Y. Fischer, M. Frahm, E. Garutti, A. Grohsjean, A. A. Guvenli, J. Haller, D. Hundhausen, G. Kasieczka, P. Keicher, R. Klanner, W. Korcari, T. Kramer, C. C. Kuo, F. Labe, J. Lange, A. Lobanov, L. Moureaux, A. Nigamova, K. Nikolopoulos, A. Paasch, K. J. Pena Rodriguez, N. Prouvost, T. Quadfasel, B. Raciti, M. Rieger, D. Savoiu, P. Schleper, M. Schröder, J. Schwandt, M. Sommerhalder, H. Stadie, G. Steinbrück, R. Ward, B. Wiederspan, M. Wolf, S. Brommer, E. Butz, Y. M. Chen, T. Chwalek, A. Dierlamm, G. G. Dincer, U. Elicabuk, N. Faltermann, M. Giffels, A. Gottmann, F. Hartmann, R. Hofsaess, M. Horzela, F. Hummer, U. Husemann, J. Kieseler, M. Klute, R. Kunnilan Muhammed Rafeek, O. Lavoryk, J. M. Lawhorn, A. Lintuluoto, S. Maier, M. Mormile, Th. Müller, E. Pfeffer, M. Presilla, G. Quast, K. Rabbertz, B. Regnery, R. Schmieder, N. Shadskiy, I. Shvetsov, H. J. Simonis, L. Sowa, L. Stockmeier, K. Tauqeer, M. Toms, B. Topko, N. Trevisani, C. Verstege, T. Voigtländer, R. F. Von Cube, J. Von Den Driesch, M. Wassmer, R. Wolf, W. D. Zeuner, X. Zuo, G. Anagnostou, G. Daskalakis, A. Kyriakis, G. Melachroinos, Z. Painesis, I. Paraskevas, N. Saoulidou, K. Theofilatos, E. Tziaferi, E. Tzovara, K. Vellidis, I. Zisopoulos, T. Chatzistavrou, G. Karapostoli, K. Kousouris, E. Siamarkou, G. Tsipolitis, I. Bestintzanos, I. Evangelou, C. Foudas, P. Katsoulis, P. Kokkas, P. G. Kosmoglou Kioseoglou, N. Manthos, I. Papadopoulos, J. Strologas, D. Druzhkin, C. Hajdu, D. Horvath, K. Márton, A. J. Rádl, F. Sikler, V. Veszpremi, M. Csanád, K. Farkas, A. Fehérkuti, M. M. A. Gadallah, Á. Kadlecsik, M. León Coello, G. Pásztor, G. I. Veres, B. Ujvari, G. Zilizi, G. Bencze, S. Czellar, J. Molnar, Z. Szillasi, T. Csorgo, F. Nemes, T. Novak, I. Szanyi, S. Bansal, S. B. Beri, V. Bhatnagar, G. Chaudhary, S. Chauhan, N. Dhingra, A. Kaur, A. Kaur, H. Kaur, M. Kaur, S. Kumar, T. Sheokand, J. B. Singh, A. Singla, A. Bhardwaj, A. Chhetri, B. C. Choudhary, A. Kumar, A. Kumar, M. Naimuddin, S. Phor, K. Ranjan, M. K. Saini, S. Acharya, B. Gomber, B. Sahu, S. Mukherjee, S. Baradia, S. Bhattacharya, S. Das Gupta, S. Dutta, S. Dutta, S. Sarkar, M. M. Ameen, P. K. Behera, S. Chatterjee, G. Dash, A. Dattamunsi, P. Jana, P. Kalbhor, S. Kamble, J. R. Komaragiri, T. Mishra, P. R. Pujahari, A. K. Sikdar, R. K. Singh, P. Verma, S. Verma, A. Vijay, B. K. Sirasva, L. Bhatt, S. Dugad, G. B. Mohanty, M. Shelake, P. Suryadevara, A. Bala, S. Banerjee, S. Barman, R. M. Chatterjee, M. Guchait, Sh. Jain, A. Jaiswal, B. M. Joshi, S. Kumar, M. Maity, G. Majumder, K. Mazumdar, S. Parolia, R. Saxena, A. Thachayath, S. Bahinipati, D. Maity, P. Mal, K. Naskar, A. Nayak, S. Nayak, K. Pal, R. Raturi, P. Sadangi, S. K. Swain, S. Varghese, D. Vats, A. Alpana, S. Dube, P. Hazarika, B. Kansal, A. Laha, R. Sharma, S. Sharma, K. Y. Vaish, S. Ghosh, H. Bakhshiansohi, A. Jafari, V. Sedighzadeh Dalavi, M. Zeinali, S. Bashiri, S. Chenarani, S. M. Etesami, Y. Hosseini, M. Khakzad, E. Khazaie, M. Mohammadi Najafabadi, S. Tizchang, M. Felcini, M. Grunewald, M. Abbrescia, M. Barbieri, M. Buonsante, A. Colaleo, D. Creanza, N. De Filippis, M. De Palma, W. Elmetenawee, N. Ferrara, L. Fiore, L. Longo, M. Louka, G. Maggi, M. Maggi, I. Margjeka, V. Mastrapasqua, S. My, F. Nenna, S. Nuzzo, A. Pellecchia, A. Pompili, G. Pugliese, R. Radogna, D. Ramos, A. Ranieri, L. Silvestris, F. M. Simone, Ü. Sözbilir, A. Stamerra, D. Troiano, R. Venditti, P. Verwilligen, A. Zaza, G. Abbiendi, C. Battilana, D. Bonacorsi, P. Capiluppi, F. R. Cavallo, M. Cuffiani, T. Diotalevi, F. Fabbri, A. Fanfani, D. Fasanella, P. Giacomelli, C. Grandi, L. Guiducci, S. Lo Meo, M. Lorusso, L. Lunerti, S. Marcellini, G. Masetti, F. L. Navarria, G. Paggi, A. Perrotta, F. Primavera, A. M. Rossi, S. Rossi Tisbeni, T. Rovelli, G. P. Siroli, S. Costa, A. Di Mattia, A. Lapertosa, R. Potenza, A. Tricomi, J. Altork, P. Assiouras, G. Barbagli, G. Bardelli, M. Bartolini, A. Calandri, B. Camaiani, A. Cassese, R. Ceccarelli, V. Ciulli, C. Civinini, R. D’Alessandro, L. Damenti, E. Focardi, T. Kello, G. Latino, P. Lenzi, M. Lizzo, M. Meschini, S. Paoletti, A. Papanastassiou, G. Sguazzoni, L. Viliani, L. Benussi, S. Bianco, S. Meola, D. Piccolo, M. Alves Gallo Pereira, F. Ferro, E. Robutti, S. Tosi, A. Benaglia, F. Brivio, V. Camagni, F. Cetorelli, F. De Guio, M. E. Dinardo, P. Dini, S. Gennai, R. Gerosa, A. Ghezzi, P. Govoni, L. Guzzi, M. R. Kim, G. Lavizzari, M. T. Lucchini, M. Malberti, S. Malvezzi, A. Massironi, D. Menasce, L. Moroni, M. Paganoni, S. Palluotto, D. Pedrini, A. Perego, G. Pizzati, S. Ragazzi, T. Tabarelli de Fatis, S. Buontempo, C. Di Fraia, F. Fabozzi, L. Favilla, A. O. M. Iorio, L. Lista, P. Paolucci, B. Rossi, P. Azzi, N. Bacchetta, D. Bisello, P. Bortignon, G. Bortolato, A. C. M. Bulla, R. Carlin, P. Checchia, T. Dorigo, F. Gasparini, U. Gasparini, S. Giorgetti, E. Lusiani, M. Margoni, F. Montecassiano, J. Pazzini, P. Ronchese, R. Rossin, F. Simonetto, M. Tosi, A. Triossi, S. Ventura, P. Zotto, A. Zucchetta, G. Zumerle, A. Braghieri, S. Calzaferri, P. Montagna, M. Pelliccioni, V. Re, C. Riccardi, P. Salvini, I. Vai, P. Vitulo, S. Ajmal, M. E. Ascioti, G. M. Bilei, C. Carrivale, D. Ciangottini, L. Della Penna, L. Fanò, V. Mariani, M. Menichelli, F. Moscatelli, A. Rossi, A. Santocchia, D. Spiga, T. Tedeschi, C. Aimè, C. A. Alexe, P. Asenov, P. Azzurri, G. Bagliesi, R. Bhattacharya, L. Bianchini, T. Boccali, E. Bossini, D. Bruschini, L. Calligaris, R. Castaldi, F. Cattafesta, M. A. Ciocci, M. Cipriani, R. Dell’Orso, S. Donato, R. Forti, A. Giassi, F. Ligabue, A. C. Marini, D. Matos Figueiredo, A. Messineo, S. Mishra, V. K. Muraleedharan Nair Bindhu, S. Nandan, F. Palla, M. Riggirello, A. Rizzi, G. Rolandi, S. Roy Chowdhury, T. Sarkar, A. Scribano, P. Solanki, P. Spagnolo, F. Tenchini, R. Tenchini, G. Tonelli, N. Turini, F. Vaselli, A. Venturi, P. G. Verdini, P. Akrap, C. Basile, S. C. Behera, F. Cavallari, L. Cunqueiro Mendez, F. De Riggi, D. Del Re, E. Di Marco, M. Diemoz, F. Errico, L. Frosina, R. Gargiulo, B. Harikrishnan, F. Lombardi, E. Longo, L. Martikainen, J. Mijuskovic, G. Organtini, N. Palmeri, R. Paramatti, C. Quaranta, S. Rahatlou, C. Rovelli, F. Santanastasio, L. Soffi, V. Vladimirov, N. Amapane, R. Arcidiacono, S. Argiro, M. Arneodo, N. Bartosik, R. Bellan, A. Bellora, C. Biino, C. Borca, N. Cartiglia, M. Costa, R. Covarelli, N. Demaria, L. Finco, M. Grippo, B. Kiani, L. Lanteri, F. Legger, F. Luongo, C. Mariotti, S. Maselli, A. Mecca, L. Menzio, P. Meridiani, E. Migliore, M. Monteno, M. M. Obertino, G. Ortona, L. Pacher, N. Pastrone, M. Ruspa, F. Siviero, V. Sola, A. Solano, A. Staiano, C. Tarricone, D. Trocino, G. Umoret, E. Vlasov, R. White, J. Babbar, S. Belforte, V. Candelise, M. Casarsa, F. Cossutti, K. De Leo, G. Della Ricca, R. Delli Gatti, S. Dogra, J. Hong, J. Kim, T. Kim, D. Lee, H. Lee, J. Lee, S. W. Lee, C. S. Moon, Y. D. Oh, S. Sekmen, B. Tae, Y. C. Yang, M. S. Kim, G. Bak, P. Gwak, H. Kim, D. H. Moon, J. Seo, E. Asilar, F. Carnevali, J. Choi, T. J. Kim, Y. Ryou, S. Ha, S. Han, B. Hong, J. Kim, K. Lee, K. S. Lee, S. Lee, J. Yoo, J. Goh, J. Shin, S. Yang, Y. Kang, H. S. Kim, Y. Kim, S. Lee, J. Almond, J. H. Bhyun, J. Choi, J. Choi, W. Jun, H. Kim, J. Kim, T. Kim, Y. Kim, Y. W. Kim, S. Ko, H. Lee, J. Lee, J. Lee, B. H. Oh, S. B. Oh, J. Shin, U. K. Yang, I. Yoon, W. Jang, D. Y. Kang, D. Kim, S. Kim, B. Ko, J. S. H. Lee, Y. Lee, I. C. Park, Y. Roh, I. J. Watson, G. Cho, K. Hwang, B. Kim, S. Kim, K. Lee, H. D. Yoo, M. Choi, Y. Lee, I. Yu, T. Beyrouthy, Y. Gharbia, F. Alazemi, K. Dreimanis, O. M. Eberlins, A. Gaile, C. Munoz Diaz, D. Osite, G. Pikurs, R. Plese, A. Potrebko, M. Seidel, D. Sidiropoulos Kontos, N. R. Strautnieks, M. Ambrozas, A. Juodagalvis, S. Nargelas, A. Rinkevicius, G. Tamulaitis, I. Yusuff, Z. Zolkapli, J. F. Benitez, A. Castaneda Hernandez, A. Cota Rodriguez, L. E. Cuevas Picos, H. A. Encinas Acosta, L. G. Gallegos Maríñez, J. A. Murillo Quijada, L. Valencia Palomo, G. Ayala, H. Castilla-Valdez, H. Crotte Ledesma, R. Lopez-Fernandez, J. Mejia Guisao, R. Reyes-Almanza, A. Sánchez Hernández, C. Oropeza Barrera, D. L. Ramirez Guadarrama, M. Ramírez García, I. Bautista, F. E. Neri Huerta, I. Pedraza, H. A. Salazar Ibarguen, C. Uribe Estrada, I. Bubanja, N. Raicevic, P. H. Butler, A. Ahmad, M. I. Asghar, A. Awais, M. I. M. Awan, W. A. Khan, V. Avati, L. Forthomme, L. Grzanka, M. Malawski, K. Piotrzkowski, M. Bluj, M. Górski, M. Kazana, M. Szleper, P. Zalewski, K. Bunkowski, K. Doroba, A. Kalinowski, M. Konecki, J. Krolikowski, A. Muhammad, P. Fokow, K. Pozniak, W. Zabolotny, M. Araujo, D. Bastos, C. Beirão Da Cruz E Silva, A. Boletti, M. Bozzo, T. Camporesi, G. Da Molin, M. Gallinaro, J. Hollar, N. Leonardo, G. B. Marozzo, A. Petrilli, M. Pisano, J. Seixas, J. Varela, J. W. Wulff, P. Adzic, L. Markovic, P. Milenovic, V. Milosevic, D. Devetak, M. Dordevic, J. Milosevic, L. Nadderd, V. Rekovic, M. Stojanovic, M. Alcalde Martinez, J. Alcaraz Maestre, Cristina F. Bedoya, J. A. Brochero Cifuentes, Oliver M. Carretero, M. Cepeda, M. Cerrada, N. Colino, J. Cuchillo Ortega, B. De La Cruz, A. Delgado Peris, A. Escalante Del Valle, D. Fernández Del Val, J. P. Fernández Ramos, J. Flix, M. C. Fouz, M. Gonzalez Hernandez, O. Gonzalez Lopez, S. Goy Lopez, J. M. Hernandez, M. I. Josa, J. Llorente Merino, C. Martin Perez, E. Martin Viscasillas, D. Moran, C. M. Morcillo Perez, R. Paz Herrera, C. Perez Dengra, A. Pérez-Calero Yzquierdo, J. Puerta Pelayo, I. Redondo, J. Vazquez Escobar, J. F. de Trocóniz, B. Alvarez Gonzalez, J. Ayllon Torresano, A. Cardini, J. Cuevas, J. Del Riego Badas, D. Estrada Acevedo, J. Fernandez Menendez, S. Folgueras, I. Gonzalez Caballero, P. Leguina, M. Obeso Menendez, E. Palencia Cortezon, J. Prado Pico, A. Soto Rodríguez, C. Vico Villalba, P. Vischia, S. Blanco Fernández, I. J. Cabrillo, A. Calderon, J. Duarte Campderros, M. Fernandez, G. Gomez, C. Lasaosa García, R. Lopez Ruiz, C. Martinez Rivero, P. Martinez Ruiz del Arbol, F. Matorras, P. Matorras Cuevas, E. Navarrete Ramos, J. Piedra Gomez, C. Quintana San Emeterio, L. Scodellaro, I. Vila, R. Vilar Cortabitarte, J. M. Vizan Garcia, B. Kailasapathy, D. D. C. Wickramarathna, W. G. D. Dharmaratna, K. Liyanage, N. Perera, D. Abbaneo, C. Amendola, R. Ardino, E. Auffray, J. Baechler, D. Barney, J. Bendavid, M. Bianco, A. Bocci, L. Borgonovi, C. Botta, A. Bragagnolo, C. E. Brown, C. Caillol, G. Cerminara, P. Connor, D. d’Enterria, A. Dabrowski, A. David, A. De Roeck, M. M. Defranchis, M. Deile, M. Dobson, P. J. Fernández Manteca, W. Funk, A. Gaddi, S. Giani, D. Gigi, K. Gill, F. Glege, M. Glowacki, A. Gruber, J. Hegeman, J. K. Heikkilä, B. Huber, V. Innocente, T. James, P. Janot, O. Kaluzinska, O. Karacheban, G. Karathanasis, S. Laurila, P. Lecoq, C. Lourenço, A.-M. Lyon, M. Magherini, L. Malgeri, M. Mannelli, A. Mehta, F. Meijers, J. A. Merlin, S. Mersi, E. Meschi, M. Migliorini, F. Monti, F. Moortgat, M. Mulders, M. Musich, I. Neutelings, S. Orfanelli, F. Pantaleo, M. Pari, G. Petrucciani, A. Pfeiffer, M. Pierini, M. Pitt, H. Qu, D. Rabady, A. Reimers, B. Ribeiro Lopes, F. Riti, P. Rosado, M. Rovere, H. Sakulin, R. Salvatico, S. Sanchez Cruz, S. Scarfi, M. Selvaggi, A. Sharma, K. Shchelina, P. Silva, P. Sphicas, A. G. Stahl Leiton, A. Steen, S. Summers, D. Treille, P. Tropea, E. Vernazza, J. Wanczyk, J. Wang, S. Wuchterl, M. Zarucki, P. Zehetner, P. Zejdl, G. Zevi Della Porta, T. Bevilacqua, L. Caminada, W. Erdmann, R. Horisberger, Q. Ingram, H. C. Kaestli, D. Kotlinski, C. Lange, U. Langenegger, L. Noehte, T. Rohe, A. Samalan, T. K. Aarrestad, M. Backhaus, G. Bonomelli, C. Cazzaniga, K. Datta, P. De Bryas Dexmiers D’archiacchiac, A. De Cosa, G. Dissertori, M. Dittmar, M. Donegà, F. Eble, K. Gedia, F. Glessgen, C. Grab, N. Härringer, T. G. Harte, W. Lustermann, M. Malucchi, R. A. Manzoni, M. Marchegiani, L. Marchese, A. Mascellani, F. Nessi-Tedaldi, F. Pauss, V. Perovic, B. Ristic, R. Seidita, J. Steggemann, A. Tarabini, D. Valsecchi, R. Wallny, C. Amsler, P. Bärtschi, F. Bilandzija, M. F. Canelli, G. Celotto, K. Cormier, M. Huwiler, W. Jin, A. Jofrehei, B. Kilminster, T. H. Kwok, S. Leontsinis, V. Lukashenko, A. Macchiolo, F. Meng, M. Missiroli, J. Motta, P. Robmann, M. Senger, E. Shokr, F. Stäger, R. Tramontano, P. Viscone, D. Bhowmik, C. M. Kuo, P. K. Rout, S. Taj, P. C. Tiwari, L. Ceard, K. F. Chen, Z. g. Chen, A. De Iorio, W.-S. Hou, T. H. Hsu, Y. W. Kao, S. Karmakar, G. Kole, Y. Y. Li, R.-S. Lu, E. Paganis, X. F. Su, J. Thomas-Wilsker, L. S. Tsai, D. Tsionou, H. Y. Wu, E. Yazgan, C. Asawatangtrakuldee, N. Srimanobhas, Y. Maghrbi, D. Agyel, F. Dolek, I. Dumanoglu, Y. Guler, E. Gurpinar Guler, C. Isik, O. Kara, A. Kayis Topaksu, Y. Komurcu, G. Onengut, K. Ozdemir, B. Tali, U. G. Tok, E. Uslan, I. S. Zorbakir, M. Yalvac, B. Akgun, I. O. Atakisi, E. Gülmez, M. Kaya, O. Kaya, M. A. Sarkisla, S. Tekten, A. Cakir, K. Cankocak, S. Sen, O. Aydilek, B. Hacisahinoglu, I. Hos, B. Kaynak, S. Ozkorucuklu, O. Potok, H. Sert, C. Simsek, C. Zorbilmez, S. Cerci, B. Isildak, E. Simsek, D. Sunar Cerci, T. Yetkin, A. Boyaryntsev, O. Dadazhanova, B. Grynyov, L. Levchuk, J. J. Brooke, A. Bundock, F. Bury, E. Clement, D. Cussans, D. Dharmender, H. Flacher, J. Goldstein, H. F. Heath, M.-L. Holmberg, L. Kreczko, S. Paramesvaran, L. Robertshaw, M. S. Sanjrani, J. Segal, V. J. Smith, A. H. Ball, K. W. Bell, A. Belyaev, C. Brew, R. M. Brown, D. J. A. Cockerill, A. Elliot, K. V. Ellis, J. Gajownik, K. Harder, S. Harper, J. Linacre, K. Manolopoulos, M. Moallemi, D. M. Newbold, E. Olaiya, D. Petyt, T. Reis, A. R. Sahasransu, G. Salvi, T. Schuh, C. H. Shepherd-Themistocleous, I. R. Tomalin, K. C. Whalen, T. Williams, I. Andreou, R. Bainbridge, P. Bloch, O. Buchmuller, C. A. Carrillo Montoya, D. Colling, J. S. Dancu, I. Das, P. Dauncey, G. Davies, M. Della Negra, S. Fayer, G. Fedi, G. Hall, H. R. Hoorani, A. Howard, G. Iles, C. R. Knight, P. Krueper, J. Langford, K. H. Law, J. León Holgado, E. Leutgeb, L. Lyons, A.-M. Magnan, B. Maier, S. Mallios, A. Mastronikolis, M. Mieskolainen, J. Nash, M. Pesaresi, P. B. Pradeep, B. C. Radburn-Smith, A. Richards, A. Rose, L. Russell, K. Savva, C. Seez, R. Shukla, A. Tapper, K. Uchida, G. P. Uttley, T. Virdee, M. Vojinovic, N. Wardle, D. Winterbottom, J. E. Cole, A. Khan, P. Kyberd, I. D. Reid, S. Abdullin, A. Brinkerhoff, E. Collins, M. R. Darwish, J. Dittmann, K. Hatakeyama, V. Hegde, J. Hiltbrand, B. McMaster, J. Samudio, S. Sawant, C. Sutantawibul, J. Wilson, J. M. Hogan, R. Bartek, A. Dominguez, S. Raj, A. E. Simsek, S. S. Yu, B. Bam, A. Buchot Perraguin, S. Campbell, R. Chudasama, S. I. Cooper, C. Crovella, G. Fidalgo, S. V. Gleyzer, A. Khukhunaishvili, K. Matchev, E. Pearson, C. U. Perez, P. Rumerio, E. Usai, R. Yi, S. Cholak, G. De Castro, Z. Demiragli, C. Erice, C. Fangmeier, C. Fernandez Madrazo, E. Fontanesi, J. Fulcher, F. Golf, S. Jeon, J. O’Cain, I. Reed, J. Rohlf, K. Salyer, D. Sperka, D. Spitzbart, I. Suarez, A. Tsatsos, E. Wurtz, A. G. Zecchinelli, G. Barone, G. Benelli, D. Cutts, S. Ellis, L. Gouskos, M. Hadley, U. Heintz, K. W. Ho, T. Kwon, L. Lambrecht, G. Landsberg, K. T. Lau, J. Luo, S. Mondal, J. Roloff, T. Russell, S. Sagir, X. Shen, M. Stamenkovic, N. Venkatasubramanian, S. Abbott, B. Barton, R. Breedon, H. Cai, M. Calderon De La Barca Sanchez, E. Cannaert, M. Chertok, M. Citron, J. Conway, P. T. Cox, R. Erbacher, O. Kukral, G. Mocellin, S. Ostrom, I. Salazar Segovia, J. S. Tafoya Vargas, W. Wei, S. Yoo, K. Adamidis, M. Bachtis, D. Campos, R. Cousins, A. Datta, G. Flores Avila, J. Hauser, M. Ignatenko, M. A. Iqbal, T. Lam, Y. F. Lo, E. Manca, A. Nunez Del Prado, D. Saltzberg, V. Valuev, R. Clare, J. W. Gary, G. Hanson, A. Aportela, A. Arora, J. G. Branson, S. Cittolin, S. Cooperstein, B. D’Anzi, D. Diaz, J. Duarte, L. Giannini, Y. Gu, J. Guiang, V. Krutelyov, R. Lee, J. Letts, H. Li, M. Masciovecchio, F. Mokhtar, S. Mukherjee, M. Pieri, D. Primosch, M. Quinnan, V. Sharma, M. Tadel, E. Vourliotis, F. Würthwein, A. Yagil, Z. Zhao, A. Barzdukas, L. Brennan, C. Campagnari, S. Carron Montero, K. Downham, C. Grieco, M. M. Hussain, J. Incandela, M. W. K. Lai, A. J. Li, P. Masterson, J. Richman, S. N. Santpur, U. Sarica, R. Schmitz, F. Setti, J. Sheplock, D. Stuart, T. Á. Vámi, X. Yan, D. Zhang, A. Albert, S. Bhattacharya, A. Bornheim, O. Cerri, R. Kansal, J. Mao, H. B. Newman, G. Reales Gutiérrez, T. Sievert, M. Spiropulu, J. R. Vlimant, R. A. Wynne, S. Xie, J. Alison, S. An, M. Cremonesi, V. Dutta, E. Y. Ertorer, T. Ferguson, T. A. Gómez Espinosa, A. Harilal, A. Kallil Tharayil, M. Kanemura, C. Liu, P. Meiring, T. Mudholkar, S. Murthy, P. Palit, K. Park, M. Paulini, A. Roberts, A. Sanchez, W. Terrill, J. P. Cumalat, W. T. Ford, A. Hart, A. Hassani, S. Kwan, J. Pearkes, C. Savard, N. Schonbeck, K. Stenson, K. A. Ulmer, S. R. Wagner, N. Zipper, D. Zuolo, J. Alexander, X. Chen, J. Dickinson, A. Duquette, J. Fan, X. Fan, J. Grassi, S. Hogan, P. Kotamnives, J. Monroy, G. Niendorf, M. Oshiro, J. R. Patterson, A. Ryd, J. Thom, P. Wittich, R. Zou, L. Zygala, M. Albrow, M. Alyari, O. Amram, G. Apollinari, A. Apresyan, L. A. T. Bauerdick, D. Berry, J. Berryhill, P. C. Bhat, K. Burkett, J. N. Butler, A. Canepa, G. B. Cerati, H. W. K. Cheung, F. Chlebana, C. Cosby, G. Cummings, I. Dutta, V. D. Elvira, J. Freeman, A. Gandrakota, Z. Gecse, L. Gray, D. Green, A. Grummer, S. Grünendahl, D. Guerrero, O. Gutsche, R. M. Harris, T. C. Herwig, J. Hirschauer, B. Jayatilaka, S. Jindariani, M. Johnson, U. Joshi, T. Klijnsma, B. Klima, K. H. M. Kwok, S. Lammel, C. Lee, D. Lincoln, R. Lipton, T. Liu, K. Maeshima, D. Mason, P. McBride, P. Merkel, S. Mrenna, S. Nahn, J. Ngadiuba, D. Noonan, S. Norberg, V. Papadimitriou, N. Pastika, K. Pedro, C. Pena, C. E. Perez Lara, F. Ravera, A. Reinsvold Hall, L. Ristori, M. Safdari, E. Sexton-Kennedy, N. Smith, A. Soha, L. Spiegel, S. Stoynev, J. Strait, L. Taylor, S. Tkaczyk, N. V. Tran, L. Uplegger, E. W. Vaandering, C. Wang, I. Zoi, C. Aruta, P. Avery, D. Bourilkov, P. Chang, V. Cherepanov, R. D. Field, C. Huh, E. Koenig, M. Kolosova, J. Konigsberg, A. Korytov, N. Menendez, G. Mitselmakher, K. Mohrman, A. Muthirakalayil Madhu, N. Rawal, S. Rosenzweig, V. Sulimov, Y. Takahashi, J. Wang, T. Adams, A. Al Kadhim, A. Askew, S. Bower, R. Goff, R. Hashmi, R. S. Kim, T. Kolberg, G. Martinez, M. Mazza, H. Prosper, P. R. Prova, R. Yohay, B. Alsufyani, S. Butalla, S. Das, M. Hohlmann, M. Lavinsky, E. Yanes, M. R. Adams, N. Barnett, A. Baty, C. Bennett, R. Cavanaugh, R. Escobar Franco, O. Evdokimov, C. E. Gerber, H. Gupta, M. Hawksworth, A. Hingrajiya, D. J. Hofman, J. H. Lee, C. Mills, S. Nanda, G. Nigmatkulov, B. Ozek, T. Phan, D. Pilipovic, R. Pradhan, E. Prifti, P. Roy, T. Roy, N. Singh, M. B. Tonjes, N. Varelas, M. A. Wadud, J. Yoo, M. Alhusseini, D. Blend, K. Dilsiz, O. K. Köseyan, A. Mestvirishvili, O. Neogi, H. Ogul, Y. Onel, A. Penzo, C. Snyder, E. Tiras, B. Blumenfeld, J. Davis, A. V. Gritsan, L. Kang, S. Kyriacou, P. Maksimovic, M. Roguljic, S. Sekhar, M. V. Srivastav, M. Swartz, A. Abreu, L. F. Alcerro Alcerro, J. Anguiano, S. Arteaga Escatel, P. Baringer, A. Bean, Z. Flowers, D. Grove, J. King, G. Krintiras, M. Lazarovits, C. Le Mahieu, J. Marquez, M. Murray, M. Nickel, S. Popescu, C. Rogan, C. Royon, S. Rudrabhatla, S. Sanders, C. Smith, G. Wilson, B. Allmond, N. Islam, A. Ivanov, K. Kaadze, Y. Maravin, J. Natoli, G. G. Reddy, D. Roy, G. Sorrentino, A. Baden, A. Belloni, J. Bistany-riebman, S. C. Eno, N. J. Hadley, S. Jabeen, R. G. Kellogg, T. Koeth, B. Kronheim, S. Lascio, P. Major, A. C. Mignerey, C. Palmer, C. Papageorgakis, M. M. Paranjpe, E. Popova, A. Shevelev, L. Zhang, C. Baldenegro Barrera, H. Bossi, S. Bright-Thonney, I. A. Cali, Y. C. Chen, P. C. Chou, M. D’Alfonso, J. Eysermans, C. Freer, G. Gomez-Ceballos, M. Goncharov, G. Grosso, P. Harris, D. Hoang, G. M. Innocenti, D. Kovalskyi, J. Krupa, L. Lavezzo, Y.-J. Lee, K. Long, C. Mcginn, A. Novak, M. I. Park, C. Paus, C. Reissel, C. Roland, G. Roland, S. Rothman, T. A. Sheng, G. S. F. Stephans, D. Walter, Z. Wang, B. Wyslouch, T. J. Yang, B. Crossman, W. J. Jackson, C. Kapsiak, M. Krohn, D. Mahon, J. Mans, B. Marzocchi, R. Rusack, O. Sancar, R. Saradhy, N. Strobbe, K. Bloom, D. R. Claes, G. Haza, J. Hossain, C. Joo, I. Kravchenko, A. Rohilla, J. E. Siado, W. Tabb, A. Vagnerini, A. Wightman, F. Yan, H. Bandyopadhyay, L. Hay, H. W. Hsia, I. Iashvili, A. Kalogeropoulos, A. Kharchilava, A. Mandal, M. Morris, D. Nguyen, S. Rappoccio, H. Rejeb Sfar, A. Williams, P. Young, D. Yu, G. Alverson, E. Barberis, J. Bonilla, B. Bylsma, M. Campana, J. Dervan, Y. Haddad, Y. Han, I. Israr, A. Krishna, M. Lu, N. Manganelli, R. Mccarthy, D. M. Morse, T. Orimoto, A. Parker, L. Skinnari, C. S. Thoreson, E. Tsai, D. Wood, S. Dittmer, K. A. Hahn, Y. Liu, M. Mcginnis, Y. Miao, D. G. Monk, M. H. Schmitt, A. Taliercio, M. Velasco, J. Wang, G. Agarwal, R. Band, R. Bucci, S. Castells, A. Das, A. Ehnis, R. Goldouzian, M. Hildreth, K. Hurtado Anampa, T. Ivanov, C. Jessop, A. Karneyeu, K. Lannon, J. Lawrence, N. Loukas, L. Lutton, J. Mariano, N. Marinelli, I. Mcalister, T. McCauley, C. Mcgrady, C. Moore, Y. Musienko, H. Nelson, M. Osherson, A. Piccinelli, R. Ruchti, A. Townsend, Y. Wan, M. Wayne, H. Yockey, A. Basnet, M. Carrigan, R. De Los Santos, L. S. Durkin, C. Hill, M. Joyce, M. Nunez Ornelas, D. A. Wenzl, B. L. Winer, B. R. Yates, H. Bouchamaoui, P. Das, G. Dezoort, P. Elmer, A. Frankenthal, M. Galli, B. Greenberg, N. Haubrich, K. Kennedy, G. Kopp, Y. Lai, D. Lange, A. Loeliger, D. Marlow, I. Ojalvo, J. Olsen, F. Simpson, D. Stickland, C. Tully, S. Malik, R. Sharma, S. Chandra, R. Chawla, A. Gu, L. Gutay, M. Jones, A. W. Jung, D. Kondratyev, M. Liu, G. Negro, N. Neumeister, G. Paspalaki, S. Piperov, N. R. Saha, J. F. Schulte, F. Wang, A. Wildridge, W. Xie, Y. Yao, Y. Zhong, N. Parashar, A. Pathak, E. Shumka, D. Acosta, A. Agrawal, C. Arbour, T. Carnahan, K. M. Ecklund, S. Freed, P. Gardner, F. J. M. Geurts, T. Huang, I. Krommydas, N. Lewis, W. Li, J. Lin, O. Miguel Colin, B. P. Padley, R. Redjimi, J. Rotter, M. Wulansatiti, E. Yigitbasi, Y. Zhang, O. Bessidskaia Bylund, A. Bodek, P. de Barbaro, R. Demina, A. Garcia-Bellido, H. S. Hare, O. Hindrichs, N. Parmar, P. Parygin, H. Seo, R. Taus, B. Chiarito, J. P. Chou, S. V. Clark, S. Donnelly, D. Gadkari, Y. Gershtein, E. Halkiadakis, C. Houghton, D. Jaroslawski, A. Kobert, S. Konstantinou, I. Laflotte, A. Lath, J. Martins, M. Perez Prada, B. Rand, J. Reichert, P. Saha, S. Salur, S. Schnetzer, S. Somalwar, R. Stone, S. A. Thayil, S. Thomas, J. Vora, D. Ally, A. G. Delannoy, S. Fiorendi, J. Harris, T. Holmes, A. R. Kanuganti, N. Karunarathna, J. Lawless, L. Lee, E. Nibigira, B. Skipworth, S. Spanier, D. Aebi, M. Ahmad, T. Akhter, K. Androsov, A. Bolshov, O. Bouhali, A. Cagnotta, V. D’Amante, R. Eusebi, P. Flanagan, J. Gilmore, Y. Guo, T. Kamon, S. Luo, R. Mueller, A. Safonov, N. Akchurin, J. Damgov, Y. Feng, N. Gogate, Y. Kazhykarim, K. Lamichhane, S. W. Lee, C. Madrid, A. Mankel, T. Peltola, I. Volobouev, E. Appelt, Y. Chen, S. Greene, A. Gurrola, W. Johns, R. Kunnawalkam Elayavalli, A. Melo, D. Rathjens, F. Romeo, P. Sheldon, S. Tuo, J. Velkovska, J. Viinikainen, J. Zhang, B. Cardwell, H. Chung, B. Cox, J. Hakala, G. Hamilton Ilha Machado, R. Hirosky, M. Jose, A. Ledovskoy, C. Mantilla, C. Neu, C. Ramón Álvarez, S. Bhattacharya, P. E. Karchin, A. Aravind, S. Banerjee, K. Black, T. Bose, E. Chavez, S. Dasu, P. Everaerts, C. Galloni, H. He, M. Herndon, A. Herve, C. K. Koraka, S. Lomte, R. Loveless, A. Mallampalli, A. Mohammadi, S. Mondal, T. Nelson, G. Parida, L. Pétré, D. Pinna, A. Savin, V. Shang, V. Sharma, W. H. Smith, D. Teague, H. F. Tsoi, W. Vetens, A. Warden, S. Afanasiev, V. Alexakhin, Yu. Andreev, T. Aushev, D. Budkouski, R. Chistov, M. Danilov, T. Dimova, A. Ershov, S. Gninenko, I. Gorbunov, A. Gribushin, A. Kamenev, V. Karjavine, M. Kirsanov, V. Klyukhin, O. Kodolova, V. Korenkov, I. Korsakov, A. Kozyrev, N. Krasnikov, A. Lanev, A. Malakhov, V. Matveev, A. Nikitenko, V. Palichik, V. Perelygin, S. Petrushanko, S. Polikarpov, O. Radchenko, M. Savina, V. Shalaev, S. Shmatov, S. Shulha, Y. Skovpen, K. Slizhevskiy, V. Smirnov, O. Teryaev, I. Tlisova, A. Toropin, N. Voytishin, B. S. Yuldashev, A. Zarubin, I. Zhizhin, E. Boos, V. Bunichev, M. Dubinin, V. Savrin, A. Snigirev, L. Dudko, K. Ivanov, V. Kim, V. Murzin, V. Oreshkin, D. Sosnov

**Affiliations:** 1 Yerevan Physics Institute, Yerevan, Armenia; 2 Institut für Hochenergiephysik, Vienna, Austria; 3 Universiteit Antwerpen, Antwerp, Belgium; 4 Vrije Universiteit Brussel, Brussels, Belgium; 5 Université Libre de Bruxelles, Brussels, Belgium; 6 Ghent University, Ghent, Belgium; 7 Université Catholique de Louvain, Louvain-la-Neuve, Belgium; 8 Centro Brasileiro de Pesquisas Fisicas, Rio de Janeiro, Brazil; 9 Universidade do Estado do Rio de Janeiro, Rio de Janeiro, Brazil; 10 Universidade Estadual Paulista, Universidade Federal do ABC, São Paulo, Brazil; 11 Institute for Nuclear Research and Nuclear Energy, Bulgarian Academy of Sciences, Sofia, Bulgaria; 12 University of Sofia, Sofia, Bulgaria; 13 Instituto De Alta Investigación, Universidad de Tarapacá, Casilla 7 D, Arica, Chile; 14 Universidad Tecnica Federico Santa Maria, Valparaiso, Chile; 15 Beihang University, Beijing, China; 16 Department of Physics, Tsinghua University, Beijing, China; 17 Institute of High Energy Physics, Beijing, China; 18 State Key Laboratory of Nuclear Physics and Technology, Peking University, Beijing, China; 19 State Key Laboratory of Nuclear Physics and Technology, Institute of Quantum Matter, South China Normal University, Guangzhou, China; 20 Sun Yat-Sen University, Guangzhou, China; 21 University of Science and Technology of China, Hefei, China; 22 Nanjing Normal University, Nanjing, China; 23 Institute of Modern Physics and Key Laboratory of Nuclear Physics and Ion-beam Application (MOE), Fudan University, Shanghai, China; 24 Zhejiang University, Hangzhou, Zhejiang China; 25 Universidad de Los Andes, Bogotá, Colombia; 26 Universidad de Antioquia, Medellín, Colombia; 27 Faculty of Electrical Engineering, Mechanical Engineering and Naval Architecture, University of Split, Split, Croatia; 28 Faculty of Science, University of Split, Split, Croatia; 29 Institute Rudjer Boskovic, Zagreb, Croatia; 30 University of Cyprus, Nicosia, Cyprus; 31 Charles University, Prague, Czech Republic; 32 Escuela Politecnica Nacional, Quito, Ecuador; 33 Universidad San Francisco de Quito, Quito, Ecuador; 34 Academy of Scientific Research and Technology of the Arab Republic of Egypt, Egyptian Network of High Energy Physics, Cairo, Egypt; 35 Center for High Energy Physics (CHEP-FU), Fayoum University, El-Fayoum, Egypt; 36 National Institute of Chemical Physics and Biophysics, Tallinn, Estonia; 37 Department of Physics, University of Helsinki, Helsinki, Finland; 38 Helsinki Institute of Physics, Helsinki, Finland; 39 Lappeenranta-Lahti University of Technology, Lappeenranta, Finland; 40 IRFU, CEA, Université Paris-Saclay, Gif-sur-Yvette, France; 41 Laboratoire Leprince-Ringuet, CNRS/IN2P3, Ecole Polytechnique, Institut Polytechnique de Paris, Palaiseau, France; 42 Université de Strasbourg, CNRS, IPHC UMR 7178, Strasbourg, France; 43 Centre de Calcul de l’Institut National de Physique Nucleaire et de Physique des Particules, CNRS/IN2P3, Villeurbanne, France; 44 Institut de Physique des 2 Infinis de Lyon (IP2I), Villeurbanne, France; 45 Georgian Technical University, Tbilisi, Georgia; 46 I. Physikalisches Institut, RWTH Aachen University, Aachen, Germany; 47 III. Physikalisches Institut A, RWTH Aachen University, Aachen, Germany; 48 III. Physikalisches Institut B, RWTH Aachen University, Aachen, Germany; 49 Deutsches Elektronen-Synchrotron, Hamburg, Germany; 50 University of Hamburg, Hamburg, Germany; 51 Karlsruher Institut für Technologie, Karlsruhe, Germany; 52 Institute of Nuclear and Particle Physics (INPP), NCSR Demokritos, Aghia Paraskevi, Greece; 53 National and Kapodistrian University of Athens, Athens, Greece; 54 National Technical University of Athens, Athens, Greece; 55 University of Ioánnina, Ioannina, Greece; 56 HUN-REN Wigner Research Centre for Physics, Budapest, Hungary; 57 MTA-ELTE Lendület CMS Particle and Nuclear Physics Group, Eötvös Loránd University, Budapest, Hungary; 58 Faculty of Informatics, University of Debrecen, Debrecen, Hungary; 59 HUN-REN ATOMKI-Institute of Nuclear Research, Debrecen, Hungary; 60 Karoly Robert Campus, MATE Institute of Technology, Gyongyos, Hungary; 61 Panjab University, Chandigarh, India; 62 University of Delhi, Delhi, India; 63 University of Hyderabad, Hyderabad, India; 64 Indian Institute of Technology Kanpur, Kanpur, India; 65 Saha Institute of Nuclear Physics, HBNI, Kolkata, India; 66 Indian Institute of Technology Madras, Madras, India; 67 IISER Mohali, Mohali, India; 68 Tata Institute of Fundamental Research-A, Mumbai, India; 69 Tata Institute of Fundamental Research-B, Mumbai, India; 70 National Institute of Science Education and Research, An OCC of Homi Bhabha National Institute, Bhubaneswar, Odisha India; 71 Indian Institute of Science Education and Research (IISER), Pune, India; 72 Indian Institute of Technology Hyderabad, Hyderabad, Telangana India; 73 Isfahan University of Technology, Isfahan, Iran; 74 Institute for Research in Fundamental Sciences (IPM), Tehran, Iran; 75 University College Dublin, Dublin, Ireland; 76 INFN Sezione di Bari, Università di Bari, Politecnico di Bari, Bari, Italy; 77 INFN Sezione di Bologna, Università di Bologna, Bologna, Italy; 78 INFN Sezione di Catania, Università di Catania, Catania, Italy; 79 INFN Sezione di Firenze, Università di Firenze, Florence, Italy; 80 INFN Laboratori Nazionali di Frascati, Frascati, Italy; 81 INFN Sezione di Genova, Università di Genova, Genoa, Italy; 82 INFN Sezione di Milano-Bicocca, Università di Milano-Bicocca, Milan, Italy; 83 INFN Sezione di Napoli, Università di Napoli ‘Federico II’, Naples, Italy; Università della Basilicata, Potenza, Italy, Scuola Superiore Meridionale (SSM), Naples, Italy; 84 INFN Sezione di Padova, Università di Padova, Padua, Italy, Universita degli Studi di Cagliari, Cagliari, Italy; 85 INFN Sezione di Pavia, Università di Pavia, Pavia, Italy; 86 INFN Sezione di Perugia, Università di Perugia, Perugia, Italy; 87 INFN Sezione di Pisa, Università di Pisa, Scuola Normale Superiore di Pisa, Pisa, Italy, Università di Siena, Siena, Italy; 88 INFN Sezione di Roma, Sapienza Università di Roma, Rome, Italy; 89 INFN Sezione di Torino, Università di Torino, Turin, Italy, Università del Piemonte Orientale, Novara, Italy; 90 INFN Sezione di Trieste, Università di Trieste, Trieste, Italy; 91 Kyungpook National University, Daegu, Korea; 92 Department of Mathematics and Physics-GWNU, Gangneung, Korea; 93 Institute for Universe and Elementary Particles, Chonnam National University, Kwangju, Korea; 94 Hanyang University, Seoul, Korea; 95 Korea University, Seoul, Korea; 96 Department of Physics, Kyung Hee University, Seoul, Korea; 97 Sejong University, Seoul, Korea; 98 Seoul National University, Seoul, Korea; 99 University of Seoul, Seoul, Korea; 100 Department of Physics, Yonsei University, Seoul, Korea; 101 Sungkyunkwan University, Suwon, Korea; 102 College of Engineering and Technology, American University of the Middle East (AUM), Dasman, Kuwait; 103 Department of Physics, College of Science, Kuwait University, Safat, Kuwait; 104 Riga Technical University, Riga, Latvia; 105 University of Latvia (LU), Riga, Latvia; 106 Vilnius University, Vilnius, Lithuania; 107 National Centre for Particle Physics, Universiti Malaya, Kuala Lumpur, Malaysia; 108 Universidad de Sonora (UNISON), Hermosillo, Mexico; 109 Centro de Investigacion y de Estudios Avanzados del IPN, Mexico City, Mexico; 110 Universidad Iberoamericana, Mexico City, Mexico; 111 Benemerita Universidad Autonoma de Puebla, Puebla, Mexico; 112 University of Montenegro, Podgorica, Montenegro; 113 University of Canterbury, Christchurch, New Zealand; 114 National Centre for Physics, Quaid-I-Azam University, Islamabad, Pakistan; 115 AGH University of Krakow, Kraków, Poland; 116 National Centre for Nuclear Research, Swierk, Poland; 117 Institute of Experimental Physics, Faculty of Physics, University of Warsaw, Warsaw, Poland; 118 Warsaw University of Technology, Warsaw, Poland; 119 Laboratório de Instrumentação e Física Experimental de Partículas, Lisbon, Portugal; 120 Faculty of Physics, University of Belgrade, Belgrade, Serbia; 121 VINCA Institute of Nuclear Sciences, University of Belgrade, Belgrade, Serbia; 122 Centro de Investigaciones Energéticas Medioambientales y Tecnológicas (CIEMAT), Madrid, Spain; 123 Universidad Autónoma de Madrid, Madrid, Spain; 124 Instituto Universitario de Ciencias y Tecnologías Espaciales de Asturias (ICTEA), Universidad de Oviedo, Oviedo, Spain; 125 Instituto de Física de Cantabria (IFCA), CSIC-Universidad de Cantabria, Santander, Spain; 126 University of Colombo, Colombo, Sri Lanka; 127 Department of Physics, University of Ruhuna, Matara, Sri Lanka; 128 CERN, European Organization for Nuclear Research, Geneva, Switzerland; 129 PSI Center for Neutron and Muon Sciences, Villigen, Switzerland; 130 ETH Zurich-Institute for Particle Physics and Astrophysics (IPA), Zurich, Switzerland; 131 Universität Zürich, Zurich, Switzerland; 132 National Central University, Chung-Li, Taiwan; 133 National Taiwan University (NTU), Taipei, Taiwan; 134 High Energy Physics Research Unit, Department of Physics, Faculty of Science, Chulalongkorn University, Bangkok, Thailand; 135 Tunis El Manar University, Tunis, Tunisia; 136 Physics Department, Science and Art Faculty, Çukurova University, Adana, Turkey; 137 Physics Department, Middle East Technical University, Ankara, Turkey; 138 Bogazici University, Istanbul, Turkey; 139 Istanbul Technical University, Istanbul, Turkey; 140 Istanbul University, Istanbul, Turkey; 141 Yildiz Technical University, Istanbul, Turkey; 142 Institute for Scintillation Materials of National Academy of Science of Ukraine, Kharkiv, Ukraine; 143 National Science Centre, Kharkiv Institute of Physics and Technology, Kharkiv, Ukraine; 144 University of Bristol, Bristol, UK; 145 Rutherford Appleton Laboratory, Didcot, UK; 146 Imperial College, London, UK; 147 Brunel University, Uxbridge, UK; 148 Baylor University, Waco, TX USA; 149 Bethel University, St. Paul, MN USA; 150 Catholic University of America, Washington, DC USA; 151 The University of Alabama, Tuscaloosa, AL USA; 152 Boston University, Boston, MA USA; 153 Brown University, Providence, RI USA; 154 University of California, Davis, Davis, CA USA; 155 University of California, Los Angeles, CA USA; 156 University of California, Riverside, Riverside, CA USA; 157 University of California, San Diego, La Jolla, California USA; 158 Department of Physics, University of California, Santa Barbara, Santa Barbara, CA USA; 159 California Institute of Technology, Pasadena, CA USA; 160 Carnegie Mellon University, Pittsburgh, PA USA; 161 University of Colorado Boulder, Boulder, CO USA; 162 Cornell University, Ithaca, NY USA; 163 Fermi National Accelerator Laboratory, Batavia, IL USA; 164 University of Florida, Gainesville, FL USA; 165 Florida State University, Tallahassee, FL USA; 166 Florida Institute of Technology, Melbourne, FL USA; 167 University of Illinois Chicago, Chicago, IL USA; 168 The University of Iowa, Iowa City, IA USA; 169 Johns Hopkins University, Baltimore, MD USA; 170 The University of Kansas, Lawrence, KS USA; 171 Kansas State University, Manhattan, KS USA; 172 University of Maryland, College Park, MD USA; 173 Massachusetts Institute of Technology, Cambridge, MA USA; 174 University of Minnesota, Minneapolis, MN USA; 175 University of Nebraska-Lincoln, Lincoln, NE USA; 176 State University of New York at Buffalo, Buffalo, NY USA; 177 Northeastern University, Boston, MA USA; 178 Northwestern University, Evanston, IL USA; 179 University of Notre Dame, Notre Dame, IN USA; 180 The Ohio State University, Columbus, OH USA; 181 Princeton University, Princeton, NJ USA; 182 University of Puerto Rico, Mayagüez, PR USA; 183 Purdue University, West Lafayette, IN USA; 184 Purdue University Northwest, Hammond, IN USA; 185 Rice University, Houston, TX USA; 186 University of Rochester, Rochester, NY USA; 187 Rutgers, The State University of New Jersey, Piscataway, NJ USA; 188 University of Tennessee, Knoxville, TN USA; 189 Texas A&M University, College Station, TX USA; 190 Texas Tech University, Lubbock, TX USA; 191 Vanderbilt University, Nashville, TN USA; 192 University of Virginia, Charlottesville, VA USA; 193 Wayne State University, Detroit, MI USA; 194 University of Wisconsin-Madison, Madison, WI USA; 195 Authors Affiliated with an International Laboratory Covered by a Cooperation Agreement with CERN, Geneva, Switzerland; 196 Authors Affiliated with an Institute Formerly Covered by a Cooperation Agreement with CERN, Geneva, Switzerland; 197 Yerevan State University, Yerevan, Armenia; 198 TU Wien, Vienna, Austria; 199 Ghent University, Ghent, Belgium; 200 Universidade do Estado do Rio de Janeiro, Rio de Janeiro, Brazil; 201 FACAMP-Faculdades de Campinas, São Paulo, Brazil; 202 Universidade Estadual de Campinas, Campinas, Brazil; 203 Federal University of Rio Grande do Sul, Porto Alegre, Brazil; 204 The University of the State of Amazonas, Manaus, Brazil; 205 University of Chinese Academy of Sciences, Beijing, China; 206 China Center of Advanced Science and Technology, Beijing, China; 207 University of Chinese Academy of Sciences, Beijing, China; 208 School of Physics, Zhengzhou University, Zhengzhou, China; 209 Henan Normal University, Xinxiang, China; 210 University of Shanghai for Science and Technology, Shanghai, China; 211 The University of Iowa, Iowa City, IA USA; 212 Center for High Energy Physics, Peking University, Beijing, China; 213 Helwan University, Cairo, Egypt; 214 Zewail City of Science and Technology, Zewail, Egypt; 215 British University in Egypt, Cairo, Egypt; 216 Purdue University, West Lafayette, IN USA; 217 Université de Haute Alsace, Mulhouse, France; 218 Ilia State University, Tbilisi, Georgia; 219 an Institute Formerly Covered by a Cooperation Agreement with CERN, Geneva, Switzerland; 220 University of Hamburg, Hamburg, Germany; 221 III. Physikalisches Institut A, RWTH Aachen University, Aachen, Germany; 222 Bergische University Wuppertal (BUW), Wuppertal, Germany; 223 Brandenburg University of Technology, Cottbus, Germany; 224 Forschungszentrum Jülich, Jülich, Germany; 225 CERN, European Organization for Nuclear Research, Geneva, Switzerland; 226 HUN-REN ATOMKI-Institute of Nuclear Research, Debrecen, Hungary; 227 Universitatea Babes-Bolyai-Facultatea de Fizica, Cluj-Napoca, Romania; 228 MTA-ELTE Lendület CMS Particle and Nuclear Physics Group, Eötvös Loránd University, Budapest, Hungary; 229 HUN-REN Wigner Research Centre for Physics, Budapest, Hungary; 230 Physics Department, Faculty of Science, Assiut University, Asyût, Egypt; 231 The University of Kansas, Lawrence, KS USA; 232 Punjab Agricultural University, Ludhiana, India; 233 University of Hyderabad, Hyderabad, India; 234 Indian Institute of Science (IISc), Bangalore, India; 235 University of Visva-Bharati, Santiniketan, India; 236 IIT Bhubaneswar, Bhubaneswar, India; 237 Institute of Physics, Bhubaneswar, India; 238 Deutsches Elektronen-Synchrotron, Hamburg, Germany; 239 Isfahan University of Technology, Isfahan, Iran; 240 Sharif University of Technology, Tehran, Iran; 241 Department of Physics, University of Science and Technology of Mazandaran, Behshahr, Iran; 242 Department of Physics, Faculty of Science, Arak University, Arak, Iran; 243 Italian National Agency for New Technologies, Energy and Sustainable Economic Development, Bologna, Italy; 244 Centro Siciliano di Fisica Nucleare e di Struttura Della Materia, Catania, Italy; 245 Università degli Studi Guglielmo Marconi, Rome, Italy; 246 Scuola Superiore Meridionale, Università di Napoli ‘Federico II’, Naples, Italy; 247 Fermi National Accelerator Laboratory, Batavia, IL USA; 248 Lulea University of Technology, Luleå, Sweden; 249 Consiglio Nazionale delle Ricerche-Istituto Officina dei Materiali, Perugia, Italy; 250 UPES-University of Petroleum and Energy Studies, Dehra Dun, India; 251 Institut de Physique des 2 Infinis de Lyon (IP2I ), Villeurbanne, France; 252 Department of Applied Physics, Faculty of Science and Technology, Universiti Kebangsaan Malaysia, Bangi, Malaysia; 253 Trincomalee Campus, Eastern University, Sri Lanka, Nilaveli, Sri Lanka; 254 Saegis Campus, Nugegoda, Sri Lanka; 255 National and Kapodistrian University of Athens, Athens, Greece; 256 Ecole Polytechnique Fédérale Lausanne, Lausanne, Switzerland; 257 Universität Zürich, Zurich, Switzerland; 258 Stefan Meyer Institute for Subatomic Physics, Vienna, Austria; 259 Research Center of Experimental Health Science, Near East University, Mersin, Turkey; 260 Konya Technical University, Konya, Turkey; 261 Izmir Bakircay University, Izmir, Turkey; 262 Adiyaman University, Adiyaman, Turkey; 263 Bozok Universitetesi Rektörlügü, Yozgat, Turkey; 264 Istanbul Sabahattin Zaim University, Istanbul, Turkey; 265 Marmara University, Istanbul, Turkey; 266 Milli Savunma University, Istanbul, Turkey; 267 Informatics and Information Security Research Center, Gebze/Kocaeli, Turkey; 268 Kafkas University, Kars, Turkey; 269 Istanbul Okan University, Istanbul, Turkey; 270 Hacettepe University, Ankara, Turkey; 271 Erzincan Binali Yildirim University, Erzincan, Turkey; 272 Faculty of Engineering, Istanbul University-Cerrahpasa, Istanbul, Turkey; 273 Yildiz Technical University, Istanbul, Turkey; 274 Istinye University, Istanbul, Turkey; 275 School of Physics and Astronomy, University of Southampton, Southampton, UK; 276 Faculty of Science, Monash University, Clayton, Australia; 277 Bethel University, St. Paul, MN USA; 278 Università di Torino, Turin, Italy; 279 Karamanoğlu Mehmetbey University, Karaman, Turkey; 280 California Lutheran University, Thousand Oaks, CA USA; 281 California Institute of Technology, Pasadena, CA USA; 282 United States Naval Academy, Annapolis, MD USA; 283 Bingol University, Bingol, Turkey; 284 Georgian Technical University, Tbilisi, Georgia; 285 Sinop University, Sinop, Turkey; 286 Erciyes University, Kayseri, Turkey; 287 Horia Hulubei National Institute of Physics and Nuclear Engineering (IFIN-HH), Bucharest, Romania; 288 Another Institute Formerly Covered by a Cooperation Agreement with CERN, Geneva, Switzerland; 289 Hamad Bin Khalifa University (HBKU), Doha, Qatar; 290 Another Institute Formerly Covered by a Cooperation Agreement with CERN, Geneva, Switzerland; 291 Yerevan Physics Institute, Yerevan, Armenia; 292 Imperial College, London, UK; 293 Institute of Nuclear Physics of the Uzbekistan Academy of Sciences, Tashkent, Uzbekistan; 294 CERN, Geneva, Switzerland

## Abstract

This paper presents a search for new physics through the process where a massive particle, X, decays into a Higgs boson and a second particle, Y. The Higgs boson subsequently decays into a bottom quark–antiquark pair, which is reconstructed as a single large-radius jet. The decay products of Yare also assumed to produce a single large-radius jet. The identification of the Yparticle is enhanced by computing the anomaly score of its candidate jet using an autoencoder, which measures deviations from typical quark- or gluon-induced jets. This allows a simultaneous search for multiple Ydecay scenarios within a single analysis. In the main benchmark process, Yis a scalar particle that decays into a Wboson pair. Two other scalar Ydecay processes are also considered as benchmarks: decays to a light quark–antiquark pair, and decays to a top quark–antiquark pair. A fourth benchmark process considers Yas a hadronically decaying top quark, arising from the decay of a vector-like quark into a top quark and a Higgs boson. Data recorded by the CMS experiment at a center-of-mass energy of 13$\,\text {Te}\hspace{-.08em}\text {V}$ in 2016–2018, corresponding to an integrated luminosity of 138$\,\text {fb}^{-1}$, are analyzed. The search covers Xmasses between 1.4 and 3.0$\,\text {Te}\hspace{-.08em}\text {V}$ and Ymasses between 90 and 400$\,\text {Ge}\hspace{-.08em}\text {V}$, with all simulated signals produced in the narrow-width approximation. No significant excess above the standard model background expectation is observed. The most stringent upper limits to date are placed on benchmark signal cross sections for various masses of X and Y particles

## Introduction

The standard model (SM) of particle physics [1–3] is an exceptionally successful theory, demonstrating remarkable agreement across the whole range of experimental observations. Despite its success, there are indications that point toward the existence of physics beyond the SM (BSM). These include, among others, the unknown nature of dark matter [4], and the insufficient violation of the combined charge conjugation and parity symmetry to explain the observed baryon asymmetry of the universe [5].

To address such questions, a multitude of BSM theoretical models, such as supersymmetry [6, 7], large or warped extra dimensions [8–10], and those incorporating additional scalar or gauge particles [11] have been proposed. A common feature of these theoretical models is the prediction of new particles. Therefore, one avenue of exploration for BSM physics involves resonance searches, which aim to reconstruct the four-momentum of a new particle by analyzing its decay products. Recent advancements in machine-learning techniques have further motivated the development of inclusive, model-independent searches capable of probing multiple decay scenarios without reliance on specific assumptions [12]. Signatures involving the Higgs boson are well motivated in BSM searches, as new particles may preferentially couple to it [13, 14].

**Fig. 1:**
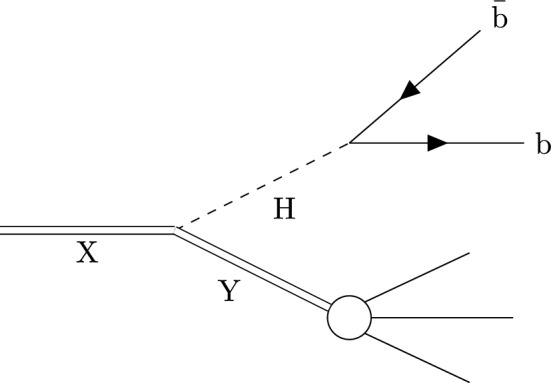
An illustration showing the signal targeted by this analysis. The final state consists of a large-radius jet originating from Hdecaying to $\text {b}\bar{\text {b}}$and another large-radius jet originating from the decay of a second particle, Y

We search for processes of the form $\textrm{X}\rightarrow \text {H}\textrm{Y},$ where Xis a heavy BSM resonance, His the 125$\,\text {Ge}\hspace{-.08em}\text {V}$ Higgs boson, and Ycan be either an SM or a BSM particle. We focus on the case where Hdecays to a bottom quark–antiquark pair ($\text {b}\bar{\text {b}}$), its most probable decay mode, and Ygives rise to a hadronic jet. The analysis targets the regime where Xis sufficiently massive that both Hand Yare produced with large Lorentz boosts, causing their decay products to be collimated and reconstructed as large-radius jets clustered with a distance parameter of 0.8 [15, 16]. An illustration of the process of interest is shown in Fig. 1. The analysis uses proton-proton ($\text {p} \text {p}$) collision data at $\sqrt{s}=13\,\text {Te}\hspace{-.08em}\text {V}$ collected by the CMS experiment at the CERN LHC in 2016–2018, and corresponding to an integrated luminosity of 138$\,\text {fb}^{-1}$.

We evaluate the results in four benchmark signal scenarios. In the main scenario, Yis a scalar particle decaying to a VVpair, where V(Wor Zboson) decays hadronically. We choose the $\textrm{W}\textrm{W}$ decay as the proxy for this process; however, similar sensitivity is expected also for the $\textrm{Z}\textrm{Z}$ or $\textrm{W}\textrm{Z}$ decays. The decay allows for one or both vector bosons to be off-shell. Two additional scalar particle decay modes are considered, with Ydecaying either into a light-flavored quark–antiquark pair ($\text {q}\bar{\text {q}}$) or a top quark–antiquark pair ($\text {t}\bar{\text {t}}$), in which both top quarks decay fully hadronically. In the fourth scenario, Yis a top quark decaying fully hadronically. These four scenarios span a range of jet substructures, enabling the evaluation of the analysis performance across diverse signal signatures. The first three benchmark scenarios are motivated by theoretical models, such as the next-to-minimal supersymmetric extension of the SM (NMSSM) [17], in which cascade decays involving more than one new particle become possible. Reference [13] provides an overview of CMS searches for the $\text {p}\text {p}\rightarrow S^{\prime }\rightarrow \text {H}+\textrm{S}$ process, where ${\textrm{S}}'$ and Srepresent new scalar particles and Sis assumed to decay to $\text {b}\bar{\text {b}}$. However, depending on the NMSSM parameters, other Sdecay modes may be favored, motivating searches targeting alternative Sdecays. The presented analysis thus complements Ref. [13] by exploring benchmark models with different Sdecay modes. The fourth signal scenario is motivated by models predicting the existence of vector-like quarks [18].

This work builds on a generic dijet anomaly search performed by the CMS Collaboration [19], but it specifies one particle and its decay mode ($\text {H}\rightarrow \text {b}\bar{\text {b}}$). This enables the application of more specific event selection criteria, enhancing the sensitivity of the analysis, while retaining a degree of generality in the characterization of the second particle.

The ParticleNet  [20] jet tagging algorithm, is applied to Hcandidate jets to assign scores indicating consistency with the decay of a massive resonance into $\text {b}\bar{\text {b}}$. The ParticleNet score is used to define “Pass” and “Fail” analysis regions. An autoencoder (AE) [19] is applied to Ycandidate jets to assign anomaly scores, with higher scores indicating deviation from jets typical of multijet production via quantum chromodynamics (QCD). This approach enables the identification of jets with unusual substructure, potentially arising from BSM decays, without relying on specific signal models. The anomaly score is used to define measurement (MR) and control (CR) analysis regions. Thus, the analysis uses four orthogonal regions, with the search performed in MR Pass. The specific selection criteria for these regions are defined in Sect. 4.

The search is carried out in a two-dimensional (2D) plane defined by the mass of the Ycandidate jet, $M_\textrm{j}^{\textrm{Y}}$, and the dijet invariant mass of the Yand Hcandidate jets, $M_\textrm{jj}$. For a signal process, the two variables correspond to the masses of Yand X, denoted as $M_{\textrm{Y}}$ and $M_{\textrm{X}}$, respectively. As a result, the analysis searches for a localized excess of events in the $M_\textrm{jj}$-$M_\textrm{j}^{\textrm{Y}}$ plane.

The paper is organized as follows. After the description of the CMS detector and event reconstruction in Sect. 2, we describe signal and background processes in Sect. 3. The event selection is discussed in Sect. 4, followed by the description of the statistical model in Sect. 5 and systematic uncertainties in Sect. 6. The results are presented in Sect. 7, followed by a summary of the paper in Sect. 8.

## The CMS detector and event reconstruction

The CMS apparatus [21] is a multipurpose, nearly hermetic detector, designed to trigger on [22–24] and identify electrons, muons, photons, and charged and neutral hadrons [25–27]. Its central feature is a superconducting solenoid of 6$\,\text {m}$ internal diameter, providing a magnetic field of 3.8$\,\text {T}$. Within the solenoid volume are a silicon pixel and strip tracker, a lead tungstate crystal electromagnetic calorimeter (ECAL), and a brass and scintillator hadron calorimeter (HCAL), each composed of a barrel and two endcap sections. Forward calorimeters extend the pseudorapidity $(\eta )$ coverage provided by the barrel and endcap detectors. Muons are reconstructed using gas-ionization detectors embedded in the steel flux-return yoke outside the solenoid. More detailed descriptions of the CMS detector, together with a definition of the coordinate system used and the relevant kinematic variables, can be found in Refs. [21, 28].

Events of interest are selected using a two-tiered trigger system. The first level, composed of custom hardware processors, uses information from the calorimeters and muon detectors to select events at a rate of around 100$\,\text {kHz}$ within a fixed latency of about 4$\,\mu \text {s}$  [22]. The second level, known as the high-level trigger, consists of a farm of processors running a version of the full event reconstruction software optimized for fast processing, and reduces the event rate to a few kHz before data storage [23, 24].

A particle-flow algorithm [29] aims to reconstruct and identify each individual particle in an event, with an optimized combination of information from the various elements of the CMS detector. The energy of photons is obtained from the ECAL measurement. The energy of electrons is determined from a combination of the electron momentum at the primary interaction vertex as determined by the tracker, the energy of the corresponding ECAL cluster, and the energy sum of all bremsstrahlung photons spatially compatible with originating from the electron track. The energy of muons is obtained from the curvature of the corresponding track. The energy of charged hadrons is determined from a combination of their momentum measured in the tracker and the matching ECAL and HCAL energy deposits, corrected for the response function of the calorimeters to hadronic showers. Finally, the energy of neutral hadrons is obtained from the corresponding corrected ECAL and HCAL energies.

For each event, hadronic jets are clustered from these reconstructed particles using the infrared- and collinear-safe anti-$k_{\textrm{T}}$ algorithm [15, 16] with a radius parameter of either 0.4 (AK4 jets) or 0.8 (AK8 jets). Jet momentum is determined as the vectorial sum of all particle momenta in the jet, and is found from simulation to be, on average, within 5–10% of the true momentum over the whole transverse momentum ($p_{\textrm{T}}$) spectrum and detector acceptance. Additional $\text {p} \text {p}$ interactions within the same or nearby bunch crossings (pileup) can contribute additional tracks and calorimetric energy depositions to the jet momentum. The pileup-per-particle identification algorithm [30, 31] is used to mitigate the effect of pileup at the reconstructed particle level, making use of local shape information, event pileup properties, and tracking information. A local shape variable is defined, which distinguishes between collinear and soft diffuse distributions of other particles surrounding the particle under consideration. The former is attributed to particles originating from the hard scatter and the latter to particles originating from pileup interactions. Charged particles identified to be originating from pileup vertices are discarded. For each neutral particle, this variable is computed using the surrounding charged particles compatible with the primary interaction vertex within the tracker acceptance $(|\eta | < 2.5),$ and using both charged and neutral particles in the region outside of the tracker coverage. The momenta of the neutral particles are then rescaled according to their probability to originate from the primary interaction vertex deduced from the local shape variable, superseding the need for jet-based pileup corrections [30].

For AK8 jets, masses are computed after applying grooming [32] techniques, which remove soft, wide-angle and collinear radiation from the jets, in order to mitigate the effects of contamination from initial-state radiation, the underlying event, and multiple hadron scattering. The trimming algorithm [33] uses a subjet size parameter of 0.3 and a radiation fraction parameter $z=0.1,$ which determines the minimum $p_{\textrm{T}}$ fraction that the reclustered jet constituents need to have in order not to be removed. The mass of the resultant jet is referred to as its “trimmed mass”. The “soft-drop mass” of the jet is obtained as follows. The constituents of the AK8 jets are reclustered using the Cambridge–Aachen algorithm [34, 35]. Then, the “modified mass drop tagger” algorithm [36, 37], also known as the “soft drop” algorithm, is applied. The algorithm is applied with angular exponent $\beta = 0,$ soft cutoff threshold $z_{\textrm{cut}} < 0.1,$ and characteristic radius $R_{0} = 0.8$ [38], which corresponds to the AK8 jet clustering distance parameter.

## Signal and background processes

The main benchmark decay mode in this paper is $\textrm{X} \rightarrow \textrm{HY}\rightarrow \text {b}\bar{\text {b}} \text {W}^{+}\text {W}^{-}$, where Xand Yare scalar particles, and where both Wbosons decay hadronically. It is simulated at leading order (LO) using the MadGraph 5_amc@nlo 2.6.5 [39] event generator and the NMSSM model [17, 40]. The samples are generated for 42 different combinations of $M_{\textrm{X}}$ and $M_{\textrm{Y}}$ values, with $M_{\textrm{X}}$ ranging from 1400 to 3000$\,\text {Ge}\hspace{-.08em}\text {V}$, and $M_{\textrm{Y}}$ ranging from 90 to 400$\,\text {Ge}\hspace{-.08em}\text {V}$. The range of $M_{\textrm{X}}$ is chosen to ensure full trigger efficiency at low mass and sufficient background event yields for the background estimation procedure at high mass, while the $M_{\textrm{Y}}$ values are selected to maintain validity of the Lorentz-boosted regime across the $M_{\textrm{X}}$ spectrum. Two other scalar decay modes, $\textrm{Y}\rightarrow \text {q}\bar{\text {q}}$ (with $\text {u}\bar{\text {u}}$ used as a proxy) and $\textrm{Y}\rightarrow \text {t}\bar{\text {t}},$ are also simulated at LO with the NMSSM model using MadGraph 5_amc@nlo 2.6.5, each with a fixed $M_{\textrm{Y}}$ (200 and 400$\text {Ge}\hspace{-.08em}\text {V}$ , respectively) and the same range of $M_{\textrm{X}}$ values as the main benchmark. The above scalar benchmark processes are modeled assuming production via gluon-gluon fusion. The $\textrm{Y}\rightarrow \text {b}\text {q}\bar{\text {q}}^\prime$ decay mode is also considered, motivated by models predicting vector-like quarks [18]. In this scenario, Xis identified as a vector-like quark and Yas the top quark. Here, the anomalous jet originates from the fully hadronic decay of the top quark, providing an example that such jets do not necessarily have to come from a BSM particle. This scenario is simulated at LO for six different $M_{\textrm{X}}$ values with $M_{\textrm{Y}}$ set to the mass of the top quark. In the rest of the paper, we differentiate between the signal models by specifying the decay mode of Y. All generated signal samples assume the narrow-width approximation for Xand Y.

The SM background consists of $\text {t}\bar{\text {t}}$production, QCD multijet events, $\textrm{W}\!+\!\text {jets}$ and $\textrm{Z}\!+\!\text {jets}$ processes, and Hproduction, with and without an associated vector boson.

The $\text {t}\bar{\text {t}}$events in all-hadronic or lepton(s)+jets final states are modeled using powheg 2.0 [41–44], at next-to-LO (NLO). The simulated $\text {t}\bar{\text {t}}$event yields are normalized using a cross section of 834$\,\text {pb}$, calculated at next-to-NLO (NNLO) in QCD with soft gluon resummation at next-to-next-to-leading logarithmic precision [45]. The theoretical uncertainty on this cross section is approximately 5%, combining scale, parton distribution function (PDF), and top mass uncertainties in quadrature.

The QCD multijet, $\textrm{W}\!+\!\text {jets}$, and $\textrm{Z}\!+\!\text {jets}$ event samples are simulated at LO using the MadGraph 5_amc@nlo event generator. These backgrounds have nonresonant distributions in the analysis observables and are thus jointly estimated using data from the sidebands. Their simulated samples are only used to develop the tools for the analysis. The gluon-gluon fusion Hboson production process is simulated using the MiNLO event generator [46]. The powheg generator [47, 48] is used to model Hproduction via vector boson fusion and associated production with a Wor Zboson at NLO accuracy.

All samples are generated using the NNPDF3.1 [49] NNLO PDFs from the lhapdf6 PDF library [50]. The showering and hadronization of partons are simulated with pythia 8.240 [51] and the CP5 underlying event tune [52].

All generated events are processed through a simulation of the CMS detector based on Geant4  [53]. The effects of pileup are modeled assuming a total inelastic $\text {p} \text {p}$ cross section of 69.2$\,\text {mb}$ [54] by overlapping simulated minimum bias events with the hard-scattering process. All simulated event samples are weighted to match the distribution of pileup vertices observed in the data.

## Event selection

The online selection uses triggers based on significant jet activity. Different trigger thresholds were used in 2016 (2017 and 2018) as follows. One trigger criterion required a single AK8 jet with $p_{\textrm{T}} >450$ (500)$\,\text {Ge}\hspace{-.08em}\text {V}$. A second trigger required that $H_{\textrm{T}}$, the scalar sum of the $p_{\textrm{T}}$ of all AK4 jets with $p_{\textrm{T}} >30\,\text {Ge}\hspace{-.08em}\text {V}$ and $|\eta | <2.5,$ must be greater than 900 (1050)$\,\text {Ge}\hspace{-.08em}\text {V}$. Additionally, in 2017–2018, a third trigger algorithm required an AK8 jet with a trimmed mass $>30$ $\,\text {Ge}\hspace{-.08em}\text {V}$ along with $p_{\textrm{T}} >400\,\text {Ge}\hspace{-.08em}\text {V}.$ The combined logical OR of all the triggers is used. The trigger selection is fully efficient with respect to the offline selection.

The offline selection requires the two leading (in $p_{\textrm{T}}$) AK8 jets to have $p_{\textrm{T}} >300\,\text {Ge}\hspace{-.08em}\text {V}$ and $|\eta | < 2.4.$ A requirement is also placed on the invariant mass of the two jets, $M_\textrm{jj} > 1300\,\text {Ge}\hspace{-.08em}\text {V},$ where $M_\textrm{jj}$ is computed from the jet four-vectors, including the jet mass. A selection is also placed on the difference in $\eta$ between the jets, $|\varDelta \eta _{\textrm{jj}} |<1.3,$ in order to further suppress the QCD multijet background, which is characterized by larger jet separation. Finally, the Hcandidate jet, defined below, is required to have its soft-drop mass in the 100–150$\,\text {Ge}\hspace{-.08em}\text {V}$ range.

The ParticleNet algorithm [55] is used to discriminate AK8 jets consistent with the decay of a massive particle into $\text {b}\bar{\text {b}}$against the background from jets originating from QCD multijet production. A mass-decorrelated version of ParticleNet  [56] is used to ensure that background-enriched regions can be defined using jets with low ParticleNet scores while maintaining the same background mass distributions in the regions passing the ParticleNet selection. The jet with the higher ParticleNet score is considered to be the Hcandidate jet and the other to be the Ycandidate jet. The soft-drop mass of the Ycandidate jet ($M_\textrm{j}^{\textrm{Y}}$) is used as a second observable alongside $M_\textrm{jj}$.

An AE neural network [57] is used to quantify the anomaly score of the Ycandidate jet. An AE consists of two parts. The first part, the “encoder”, compresses an input into a latent representation of reduced dimensionality. The second part, the “decoder”, reverses this process and attempts to reconstruct the original input from the reduced representation. We choose to represent jets as $32\times 32$ pixel images [58], covering a region of $\eta$–$\phi$ space with width $\varDelta \eta =\varDelta \phi =1.2$ centered on the jet axis, where $\phi$ is the azimuthal angle in radians. The intensity of each pixel is determined by the sum of energies from the constituent particles within the given $\eta$–$\phi$ region. We define the anomaly score as the mean squared difference between the pixel intensities of the input image and the reconstructed output. This AE architecture was previously used in Ref. [19] as part of the “Tag N’ Train” method (as discussed in Appendix A.2 of Ref. [19]). The training is performed on simulated QCD jets, allowing the AE to compress these jets with minimal information loss. However, for non-QCD jets (from signal, $\text {t}\bar{\text {t}}$production, etc.), which exhibit different substructure patterns, this procedure leads to poor compression and reconstruction of the original jet. This enables effective discrimination of non-QCD jets.

Each event is categorized into one of four groups based on the ParticleNet score of the Hcandidate jet (pass or fail) and the anomaly score of the Ycandidate jet (control region or measurement region): CR Pass, CR Fail, MR Pass, and MR Fail. The CR is used to validate the background estimation method, described below, whereas the MR is used to extract the signal. The Fail regions are used to help estimate the shape of the QCD multijet background in their corresponding Pass regions.

A ParticleNet working point corresponding to a ${\approx }60\%$ signal efficiency (defined as the fraction of signal jets passing the selection), as measured on the main benchmark with $M_{\textrm{X}} = 2000\,\text {Ge}\hspace{-.08em}\text {V}$ and $M_{\textrm{Y}} = 250\,\text {Ge}\hspace{-.08em}\text {V},$ and a ${\approx }0.5\%$ mistag rate (defined as the fraction of multijet background jets passing the selection) defines the boundary between the Pass and Fail tagging categories.

An anomaly score working point with ${\approx }85\%$ signal efficiency and 30% mistag rate on the same benchmark defines the MR region. The CR consists of jets that fail the anomaly score MR working point. A lower bound on the anomaly score is applied so that the CR remains similar to the MR, which helps with the validation of the background estimation method. As a result, about 10% of signal and 30% of QCD jets are assigned to the CR.

The ParticleNet efficiency for correctly identifying $\text {H}\rightarrow \text {b}\bar{\text {b}}$ jets is calibrated in observed data using a sample of jets originating from fragmentation of a gluon to $\text {b}\bar{\text {b}}$, which are similar to $\text {H}\rightarrow \text {b}\bar{\text {b}}$ jets. Such jets are selected from the observed data using a boosted decision tree classifier, such that their ensemble ParticleNet score resembles that of $\text {H}\rightarrow \text {b}\bar{\text {b}}$ jets [59]. A systematic uncertainty is assigned to account for the sensitivity of the results to changes in the boosted decision tree requirement applied to the jets. The data-to-simulation correction factors for the ParticleNet signal selection efficiency range from 0.93–1.05, with uncertainties up to 15%, depending on the jet $p_{\textrm{T}}$ and data-taking period. The ParticleNet efficiency for misidentifying jets from the $\text {t}\bar{\text {t}}$background as $\text {H}\rightarrow \text {b}\bar{\text {b}}$ jets is constrained directly in the fit to data by allowing the yield in the Pass and Fail regions to vary while keeping the total yield constant.

The data-to-simulation scale factors for the efficiency of the anomaly score selection are calculated using the Lund plane reweighting method [60], separately for each signal process and for the $\text {t}\bar{\text {t}}$background. The method involves applying per-jet event weights based on their distribution in the Lund plane, a 2D representation defined by the logarithms of splitting angle and $p_{\textrm{T}}$. These weights are used to adjust the simulated radiation pattern so that the density in the Lund plane matches that observed in the data. It was found that the shapes of the two observables, $M_\textrm{j}^{\textrm{Y}}$ and $M_\textrm{jj}$, do not change significantly with the application of per-jet weights. This allows applying an overall correction to the anomaly tagging efficiency instead of per-jet event reweighting. The corrections are calculated as the ratio of the anomaly tagging efficiency before and after per-jet reweighting. They are found to be compatible with unity, with 10–20% uncertainty.

## Statistical model

The analysis searches for a localized signal in the 2D $M_\textrm{jj}$-$M_\textrm{j}^{\textrm{Y}}$ plane. The $M_\textrm{jj}$ ($M_\textrm{j}^{\textrm{Y}}$) axis spans the 1300–3000 (40–500)$\,\text {Ge}\hspace{-.08em}\text {V}$ range. For higher masses, the bin widths are increased in order to ensure sufficient population of each bin. As a result, all the bins in the MR Fail have 8 or more observed events. This helps to reduce the per-bin statistical uncertainties in the QCD multijet background in the Pass region. The $M_\textrm{jj}$-$M_\textrm{j}^{\textrm{Y}}$ distributions for the background and signal predictions are simultaneously fitted to data in either the MR Pass and MR Fail, or CR Pass and CR Fail. The contributions from different data-taking periods are added together in data and simulation to construct 2D histogram templates. The templates for the $\text {t}\bar{\text {t}}$and Hproduction background processes, and for the signal benchmarks, are obtained from simulation. A pass-to-fail ratio method, described in the following paragraphs, is used to model the nonresonant background.

The pass-to-fail ratio method is based on the ratio of $M_\textrm{jj}$-$M_\textrm{j}^{\textrm{Y}}$ distributions between ParticleNet Pass and Fail regions: $R_{\mathrm {P/F}}(M_\textrm{jj},M_\textrm{j}^{\textrm{Y}})$. It is defined through the relation:

1 $$\begin{aligned} n_{\textrm{P,nr}}(i) = n_{\text {F,nr}} (i)R_{\mathrm {P/F}}(M_\textrm{jj},M_\textrm{j}^{\textrm{Y}}), \end{aligned}$$

where $n_{\mathrm {F(P),nr}}(i)$ is the number of nonresonant events in bin *i* of the $M_\textrm{jj}$-$M_\textrm{j}^{\textrm{Y}}$ distribution in the Fail (Pass) region. Since the Fail region is dominated by nonresonant events, $n_{\text {F,nr}} (i)$ can be directly estimated from data by subtracting the relatively small simulated yields of other processes. The $R_{\mathrm {P/F}}$  is a priori unknown and is determined during the fit of signal and background distributions to the data. It is modeled as a polynomial in $M_\textrm{jj}$ and $M_\textrm{j}^{\textrm{Y}}$ of order *n* and is defined as a linear combination of $M_\textrm{jj} ^a {\textrm{M}}_{\textrm{jY}}^b$ terms where the exponents *a* and *b* satisfy $a+b\le n.$ Simulation shows no strong dependence of the ParticleNet mistag rate in either of the two observables, whereas the mistag rate of anomaly tagging is observed to rise with jet mass in the simulation. This makes ParticleNet tagging the preferred method for defining the Pass and Fail regions for background estimation, ensuring that polynomials with low *n* will be able to well describe $R_{\mathrm {P/F}}$. A Fisher’s F-test [61] is used to determine the minimum polynomial order necessary and sufficient for the model. Starting from polynomials of order zero, the F-test is used to determine if the next higher order provides a significant improvement in the fit quality. The $R_{\mathrm {P/F}}$  polynomial orders in the CR and MR are discussed in Sect. 7.

**Fig. 2:**
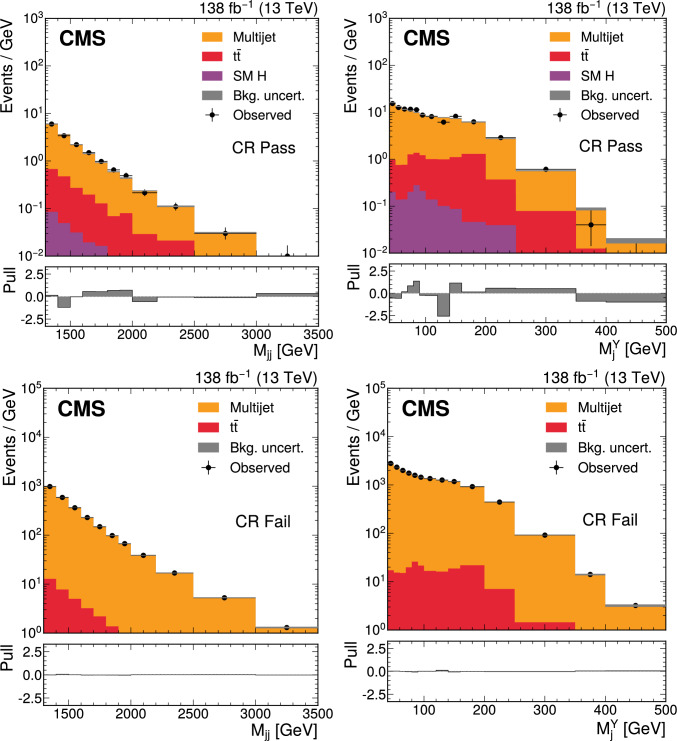
The $M_\textrm{jj}$ (left) and $M_\textrm{j}^{\textrm{Y}}$ (right) projections showing the number of observed events per $\,\text {Ge}\hspace{-.08em}\text {V}$ (black markers) compared with the backgrounds estimated in the fit to the data (filled histograms) in the CR. Pass (upper) and Fail (lower) categories are shown. The high level of agreement between the model and the data in the Fail region arises because this region is used to constrain the background estimate. The lower panels show the “Pull” defined as $(\text {observed events}{-}\text {expected events})/\sqrt{\smash [b]{\sigma _{\text {obs}} ^{2} + \sigma _{\text {bkg}} ^{2}}},$ where $\sigma _{\text {obs}}$ and $\sigma _{\text {bkg}}$ are the total uncertainties in the observation and the background estimation, respectively

**Fig. 3:**
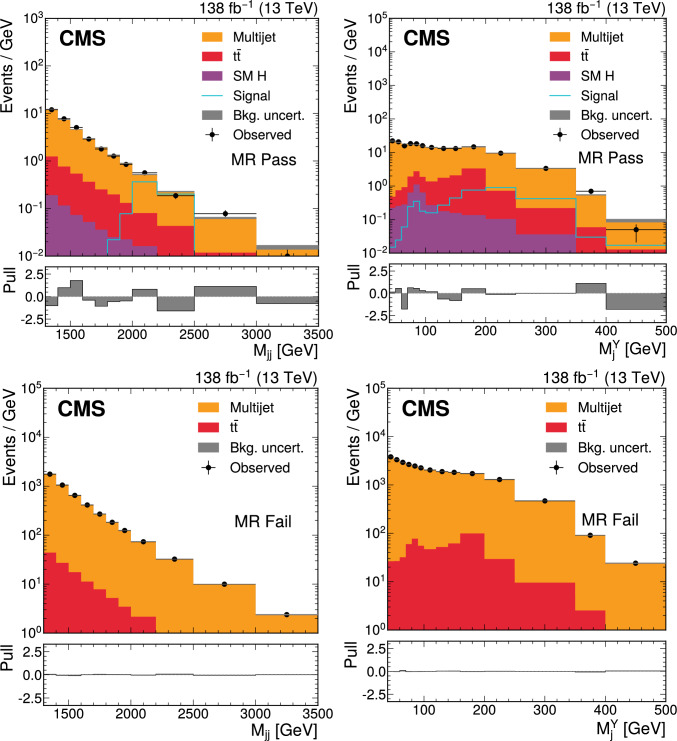
The $M_\textrm{jj}$ (left) and $M_\textrm{j}^{\textrm{Y}}$ (right) projections showing the number of observed events per $\,\text {Ge}\hspace{-.08em}\text {V}$ (black markers) compared with the backgrounds estimated in the fit to the data (filled histograms) in the MR. Pass (upper) and Fail (lower) categories are shown. The expected contribution from the signal benchmark with $M_{\textrm{X}} = 2200\,\text {Ge}\hspace{-.08em}\text {V}$ and $M_{\textrm{Y}} = 250\,\text {Ge}\hspace{-.08em}\text {V}$ is overlaid in the MR Pass, assuming a production cross section of 5$\,\text {fb}$. The high level of agreement between the model and the data in the Fail region arises because this region is used to constrain the background estimate. The lower panels show the “Pull” defined as $(\text {observed events}{-}\text {expected events})/\sqrt{\smash [b]{\sigma _{\text {obs}} ^{2} + \sigma _{\text {bkg}} ^{2}}},$ where $\sigma _{\text {obs}}$ and $\sigma _{\text {bkg}}$ are the total uncertainties in the observation and the background estimation, respectively

Systematic uncertainties, described in Sect. 6, are included in the statistical model by generating template histograms representing one standard deviation variations from the nominal simulation. These variations correspond to individual sources of uncertainty. Each is assigned a nuisance parameter in the likelihood function used for statistical interpretation, using a unit Gaussian distribution for shape uncertainties, and a log-normal distribution for normalization uncertainties [62]. These distributions change the simulated template shape according to the corresponding template uncertainty histograms.

## Systematic uncertainties

Several sources of systematic uncertainty are considered, affecting both the shapes and yields of the signal and background templates. Data-to-simulation scale factors for the ParticleNet signal and Hboson background efficiency are calculated with the method discussed in Sect. 4. They have approximately 10% uncertainty. For the $\text {t}\bar{\text {t}}$background, the mistag rate correction is directly determined during the fit using the Pass and Fail regions. The scale factors for signal and $\text {t}\bar{\text {t}}$anomaly tagging efficiency, also discussed in Sect. 4, have uncertainties of about 15–30%. For the Hboson background, a 20% uncertainty is assigned to the anomaly tagging efficiency.

Initial- and final-state radiation uncertainties are evaluated separately by varying the renormalization scale used in the parton shower by factors of 0.5 and 2. Measured uncertainties on jet energy scale and resolution are propagated to the jets used in the analysis in order to evaluate their effects. Scale and resolution uncertainties are considered uncorrelated.

The PDF uncertainties are evaluated as the square root of differences of squared PDF Hessian eigenvector sets using NNPDF3.1 PDF sets. The uncertainties coming from the choice of renormalization and factorization scales are evaluated by separately varying each of the scales while keeping the other fixed to the nominal value, and also by scaling both by factors of 0.5 and 2.0. The effect of these variations is found to be negligible for signal acceptance and thus only the $\text {t}\bar{\text {t}}$and Hboson backgrounds are affected.

A 1.6% uncertainty in the total integrated luminosity is applied [63–65]. During the 2016–2017 data taking, a gradual shift in the timing of the inputs of the ECAL hardware level trigger in the region of $|\eta | > 2$ caused a specific trigger inefficiency. Event-level weight corrections and their uncertainties are included in the simulation to account for this. A pileup correction is applied to simulated events based on the true number of interactions in the event. The corresponding uncertainty is generated by varying the inelastic $\text {p} \text {p}$ cross section at 13$\,\text {Te}\hspace{-.08em}\text {V}$ (69.2$\,\text {mb}$) by 4.6% [54]. Finally, a 5% normalization uncertainty is applied to the $\text {t}\bar{\text {t}}$cross section.

The impact of each uncertainty source on the fitted signal strength parameter is estimated by varying the corresponding nuisance parameter within its postfit uncertainty and refitting the signal strength, using the $M_{\textrm{X}} =2200\,\text {Ge}\hspace{-.08em}\text {V}$ and $M_{\textrm{Y}} =250\,\text {Ge}\hspace{-.08em}\text {V}$ simulated signal sample as a representative test case. The uncertainties with the largest impacts, ranging from 5% to 8%, are associated with the efficiency of tagging anomalous jets, the efficiency of tagging $\text {H}\rightarrow \text {b}\bar{\text {b}}$ jets, parton showering, and the renormalization and factorization scales. Other uncertainties, such as those related to the jet energy scale and resolution, integrated luminosity, and pileup, have smaller effects, altering the signal strength by 3% or less.

Overall, the sensitivity of the analysis is statistically limited, due to the limited amount of the observed data.

**Fig. 4:**
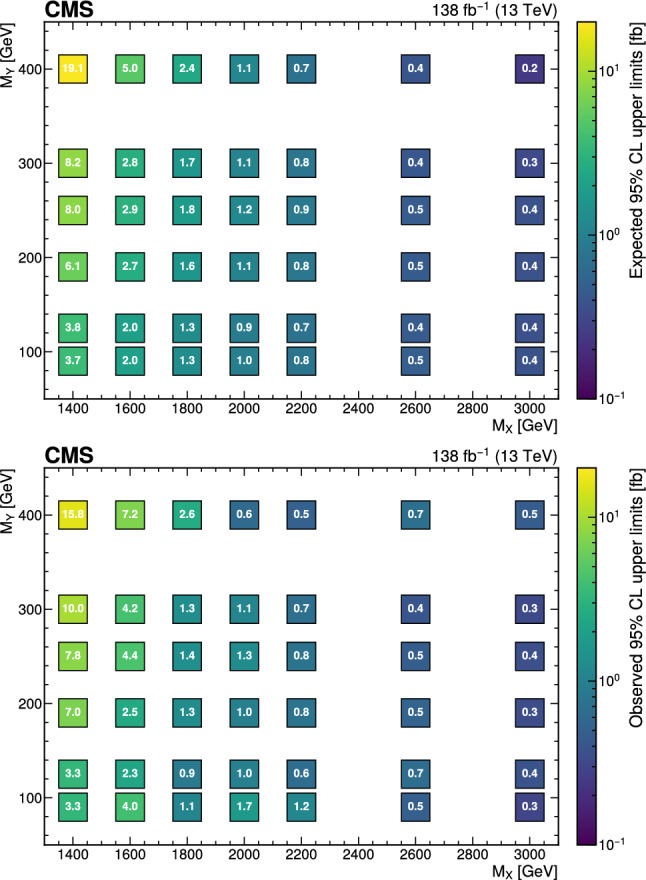
The expected (upper) and observed (lower) 95% confidence level upper limits on $\sigma (\text {p}\rightarrow \textrm{X} \rightarrow \textrm{HY}){\mathcal {B}}(\text {H}\rightarrow \text {b}\bar{\text {b}}){\mathcal {B}}(\textrm{Y}\rightarrow \textrm{W}\textrm{W} \rightarrow 2\text {q}2\bar{\text {q}}^\prime )$ for different values of $M_{\textrm{X}}$ and $M_{\textrm{Y}}$. The limits have been evaluated in discrete steps corresponding to the centers of the boxes. The numbers in the boxes are given in fb

## Results

Maximum likelihood fits are performed with the CMS statistical analysis tool Combine [62] to estimate the background and extract the fitted signal. The fits are performed separately in the CR and MR. The F-test favors an $R_{\mathrm {P/F}}$ polynomial order of $n=0\, (2)$ in the CR (MR) fit. Both fits pass the goodness-of-fit test, performed using the saturated model [66]. The *p*-value [67], indicating the level of agreement with the background-only hypothesis and the observed data, is found to be 0.59 (0.26) in the CR (MR) fit. The postfit distributions from the CR and MR fits are shown in Figs. 2 and 3, respectively.

The joint likelihoods of the signal-plus-background $M_\textrm{jj}$-$M_\textrm{j}^{\textrm{Y}}$ distributions in the MR Pass and Fail regions are constructed for different signal hypotheses. The maximum likelihood fits are used to extract the observed signal strengths. Out of the 42 $\textrm{X}\rightarrow \text {H}(\text {b}\bar{\text {b}})\textrm{Y}(\textrm{W}\textrm{W})$ mass points considered, the $M_{\textrm{X}} =1600\,\text {Ge}\hspace{-.08em}\text {V}$ and $M_{\textrm{Y}} =90\,\text {Ge}\hspace{-.08em}\text {V}$ combination corresponds to the largest local significance of 2.1 standard deviations.

**Fig. 5:**
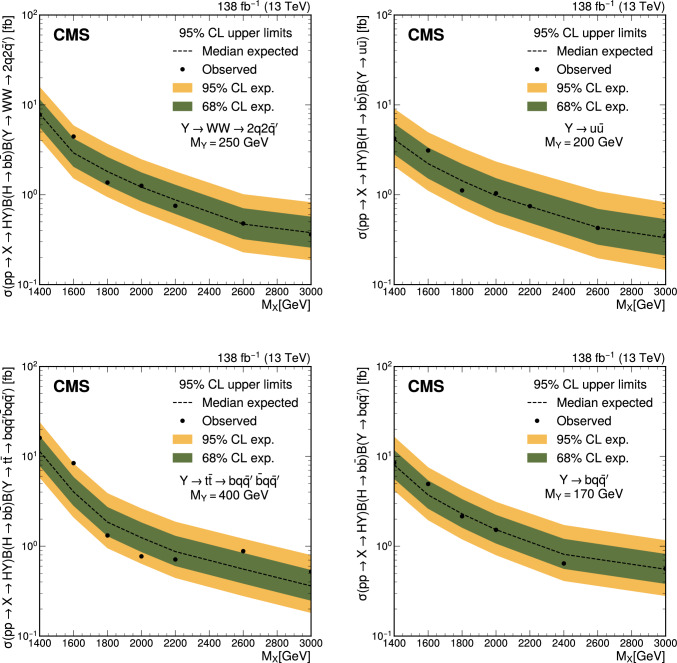
The median expected (dashed line) 95% confidence level upper limits on the main and three alternative signal scenarios as a function of $M_{\textrm{X}}$. The inner (green) and outer (yellow) bands represent the regions containing 68% and 95%, respectively, of the distribution of limits expected under the background-only hypothesis. The solid dots indicate the observed limits, which are only evaluated at the simulated mass points

The same likelihoods are used to compute upper limits on the signal cross section with a modified frequentist approach, using the $\text {CL}_\text {s}$ criterion [68, 69] with the profile likelihood ratio used as the test statistic, in an asymptotic approximation [70].

Figure 4 shows the expected and observed limits at 95% confidence level (CL), assuming the $\textrm{Y}\rightarrow \text {W}^{+}\text {W}^{-}$ decay to an all-hadronic final state. These limits range from 0.3–19.1$\,\text {fb}$. In the upper left corner of the figures, where $M_{\textrm{X}}$ is low and $M_{\textrm{Y}}$ is high, we begin to exit the Lorentz-boosted regime. This results in the Ydecay products being reconstructed as separate jets, reducing the efficiency of the offline selection and leading to higher upper limits.

Comparable searches have been performed by the ATLAS Collaboration using both unsupervised [71, 72] and weakly supervised [73] anomaly detection techniques in two-body and dijet final states. The search in Ref. [71] targets several two-body final states, while Ref. [72] specifically focuses on the $\textrm{X}\rightarrow \text {H}(\text {b}\bar{\text {b}}) \textrm{Y}$ topology, where Ydecays to a fully hadronic state, using anomalous-jet tagging. These unsupervised searches explore broader ranges of both $M_{\textrm{X}}$ and $M_{\textrm{Y}}$ than the analysis presented here, whereas the weakly supervised search [73] focuses on higher $M_{\textrm{X}}$ regions. Finally, a targeted ATLAS search for $\textrm{X}\rightarrow \text {H}\textrm{S},$ with $\text {H}\rightarrow \gamma \gamma$ and $\textrm{S}\rightarrow \text {b}\bar{\text {b}}$ [74], explores complementary lower-mass regions with $M_{\textrm{X}} < 1\,\text {Te}\hspace{-.08em}\text {V}.$ Overall, these analyses provide complementary sensitivity across different mass ranges and final-state topologies. The presented results can be compared to the CMS search for resonances decaying into two jets [19], which reports expected model-independent limits on dijet resonances in the 50–600$\,\text {fb}$ range for $M_{\textrm{X}}$ values between 1.8 and 3.0$\,\text {Te}\hspace{-.08em}\text {V}$, depending on the method and $M_{\textrm{X}}$. The most sensitive method sets limits around 60$\,\text {fb}$ across this $M_{\textrm{X}}$ range. Reference [19] also provides limits on a process that consists of a Kaluza–Klein excitation of a Wboson ($\textrm{W}_{\textrm{KK}}$) decaying into a radion and a Wboson [75, 76]. The radion then decays into two Wbosons, and all three Wbosons undergo hadronic decays. The comparison to our results is motivated by the similarity in jet substructure: both analyses involve a four-prong jet from the $\textrm{Y}\rightarrow \text {W}^{+}\text {W}^{-}$ decay and a two-prong jet originating from either Hor Wboson decay, where “prong” refers to the number of hard subjets within a large-radius jet. Although there are no directly overlapping mass points, we can compare the expected limits for a $\textrm{W}_{\textrm{KK}}$ boson mass of 3000$\,\text {Ge}\hspace{-.08em}\text {V}$ and a radion mass of 170$\,\text {Ge}\hspace{-.08em}\text {V}$ (22.1$\,\text {fb}$) with our limits for $M_{\textrm{X}} = 3000\,\text {Ge}\hspace{-.08em}\text {V}$ and $M_{\textrm{Y}} = 200\,\text {Ge}\hspace{-.08em}\text {V}$ (0.4$\,\text {fb}$). In all cases, our analysis sets more stringent limits, as expected, mostly because we require one of the jets to originate from $\text {H}\rightarrow \text {b}\bar{\text {b}}$. While this requirement makes the results less general, it enables the use of ParticleNet tagging, which provides most of the sensitivity in the analysis, with the autoencoder giving a modest additional improvement. Figure 5 shows 95% CL upper limits on the cross section for the three additional signal scenarios considered. Notably, the $\textrm{Y}\rightarrow \text {t}\bar{\text {t}}$ scenario constitutes the first search performed in the Lorentz-boosted regime for this process. The differences in the limits among the scenarios arise mainly from their distinct kinematic properties and the varying background levels in the corresponding regions of phase space, both influenced by the different assumed Ymasses.

Tabulated results of this analysis are available on HEPData [77]. The HepData entry also includes the trained AE weights used for the anomaly score evaluation.

## Summary

A search for beyond the standard model physics through the process where a resonant particle decays into a Higgs boson, H, and an additional particle Yhas been presented. The Hsubsequently decays into a bottom quark–antiquark pair, $\text {b}\bar{\text {b}}$, reconstructed as a large-radius jet in the Lorentz-boosted regime. The Hcandidate jets are tagged using the ParticleNet algorithm that is designed to recognize jets originating from a decay of a massive particle into $\text {b}\bar{\text {b}}$. The identification of the second particle, Y, is performed by computing the anomaly score of its candidate jet using an autoencoder, allowing the simultaneous search for multiple Y decay modes within a single analysis. This approach combines the targeted identification of Hdecays with model-independent anomaly detection technique, enabling a broad search for new physics. By combining the strong discrimination power of the Hboson decay to $\text {b}\bar{\text {b}}$with a model-independent anomaly detection for the second particle, this analysis achieves enhanced sensitivity for scenarios involving a Higgs boson in the decay chain, while maintaining applicability across a range of such models.

The analysis considers four benchmark models. The main benchmark assumes $\textrm{Y}\rightarrow \text {W}^{+}\text {W}^{-}$, with further hadronic decays of the Wbosons. It is simulated for $M_{\textrm{X}}$ values within 1400–3000$\,\text {Ge}\hspace{-.08em}\text {V}$ and $M_{\textrm{Y}}$ values within 90–400$\,\text {Ge}\hspace{-.08em}\text {V}$, covering 42 signal hypotheses. No significant excess above the standard model background expectations is observed. Upper limits on benchmark signal cross sections at 95% confidence level are set on the main benchmark model in the 0.3–15.8$\,\text {fb}$ range. Additionally, exclusion limits are calculated for three alternative benchmark models, assuming $\textrm{Y}\rightarrow \text {u}\bar{\text {u}}$, $\textrm{Y}\rightarrow \text {t}\bar{\text {t}}$, or $\textrm{Y}\rightarrow \text {b}\text {q}\bar{\text {q}}^\prime$ decay modes, for which the most stringent limits to date are achieved.

## Data Availability

Release and preservation of data used by the CMS Collaboration as the basis for publications is guided by the CMS data preservation, re-use and open access policy.

## Code Availability Statement

The CMS core software is publicly available on GitHub (https://github.com/cms-sw/cmssw).
